# Ecological Impact of End-of-Life-Tire (ELT)-Derived Rubbers: Acute and Chronic Effects at Organism and Population Levels

**DOI:** 10.3390/toxics10050201

**Published:** 2022-04-19

**Authors:** Stefano Magni, Erica Tediosi, Daniela Maggioni, Riccardo Sbarberi, Francesca Noé, Fabio Rossetti, Daniele Fornai, Valentina Persici, Maria Chiara Neri

**Affiliations:** 1 Department of Biosciences, University of Milan, Via Celoria 26, 20133 Milan, Italy; riccardo.sbarberi@unimi.it; 2 ChemService Controlli e Ricerche s.r.l.—Lab Analysis Group, Via Fratelli Beltrami 15, 20026 Novate Milanese, Italy; francesca.noe@chemservice.it (F.N.); mariachiara.neri@chemservice.it (M.C.N.); 3 Department of Chemistry, University of Milan, Via Golgi 19, 20133 Milan, Italy; daniela.maggioni@unimi.it; 4 Lab Analysis s.r.l., Via Europa 5, 27041 Casanova Lonati, Italy; fabio.rossetti@labanalysis.it; 5 Ecopneus scpa, Via Messina 38, 20154 Milan, Italy; d.fornai@ecopneus.it; 6 Waste and Chemicals s.r.l., Circonvallazione Gianicolense 216E, 00152 Rome, Italy; valentina.persici@wasteandchemicals.com

**Keywords:** rubbers, tire particles, acute and chronic effects, freshwater species

## Abstract

Considering the large amount of tires that reach the end of life every year, the aim of this study was the evaluation of both acute and chronic effects of end-of-life-tire (ELT)-derived rubber granules (ELT-dg) and powder (ELT-dp) on a freshwater trophic chain represented by the green alga *Pseudokirchneriella subcapitata*, the crustacean *Daphnia magna* and the teleost *Danio rerio* (zebrafish). Adverse effects were evaluated at the organism and population levels through the classical ecotoxicological tests. Acute tests on *D. magna* and *D. rerio* revealed a 50% effect concentration (EC_50_) > 100.0 mg/L for both ELT-dg and ELT-dp. Chronic exposures had a lowest observed effect concentration (LOEC) of 100.0 mg/L for both ELT-dg and ELT-dp on *P. subcapitata* grow rate and yield. LOEC decreased in the other model organisms, with a value of 9.8 mg/L for *D. magna*, referring to the number of living offspring, exposed to ELT-dg suspension. Similarly, in *D. rerio*, the main results highlighted a LOEC of 10.0 mg/L regarding the survival and juvenile weight parameters for ELT-dg and a LOEC of 10.0 mg/L concerning the survival and abnormal behavior in specimens exposed to ELT-dp. Tested materials exhibited a threshold of toxicity of 9.8 mg/L, probably a *non*-environmental concentration, although further investigations are needed to clarify the potential ecological impact of these emerging contaminants.

## 1. Introduction

The advent of plastics is unclear but could be matched between the discovery of natural rubbers by Charles Marie de La Condamine (1736) and the subsequent introduction of vulcanization by Thomas Hancock and Charles Goodyear (1843–1844), which confers to rubbers resistance for a wide plethora of uses [1]. In this context, there is not a well-defined position by the scientific community regarding the inclusion of some types of elastomers in the classification of plastics. For example, rubbers are not plastics according to the definition of the International Organization for Standardization (ISO) [2]. However, some of these materials, including tire rubbers, contain from 40 to 60% synthetic polymers (i.e., styrene-butadiene and polybutadiene rubbers) and are characterized by physical proprieties such as solid state and insolubility in water; therefore, some agencies and environmental researchers classify them among plastics [3,4,5,6]. Tires, in addition to synthetic polymers, contain silica, oil, carbon black, sulfur compounds and zinc oxide [7]. Zinc (Zn) represents 1–2% of the total weight of tires, and the leaching of this element in water represents an important environmental concern [8]. In turn, tires are considered by some authors a considerable source of plastic debris due to, e.g., their mechanical abrasion during the activity of transport [9,10,11,12,13]. This aspect originates tire-wear particles (TWPs) and tire-road-wear particles (TRWPs) when TWPs are aggregated with other road debris/pollutants [6,14]. The contribution of tires to the total amount of released plastic debris in the environment ranges from 30 to 50% in Germany, Denmark and Norway [3,9,10,15,16,17,18]. In this context, a study by Knight et al. [19] reported a concentration of these contaminants ranging from 0.6 ± 0.33 to 65 ± 7.36 particles/5 mL of analyzed material from a natural area close to a road. However, relative to studies related to the monitoring of conventional (micro)plastics, few have reported the presence of tire particles in the environment [17,20]. Indeed, the main detected plastics, e.g., in aqueous matrices, are constituted by polymers, such as polystyrene (PS), polyethylene (PE), polypropylene (PP), polyester (PEST), polyacrylate (PAK) and polyamide [21,22,23,24]. In this context, it is important to consider that tire particles are difficult to detect with the identification methodologies of conventional (micro)plastics, such as the Fourier transform infrared spectroscopy (FT-IR), due to the lack of appropriate reference standards, as well as the presence of carbon black in tires, which absorbs in the infrared region [25]. Therefore, tire particle monitoring is hampered by analytical methods, currently represented by chromatography–mass spectrometry (GC/MS) and pyrolysis GC/MS [26]. The small amounts of TWPs and TRWPs identified in monitoring studies do not correlate with the massive use of tires around the world; in Europe alone every year, about 3.4 million tons of these products reach the end of life [27]. So-called end-of-life tires (ELTs) are normally recycled into ELT-derived rubber granules (ELT-dg) or ELT-derived rubber powder (ELT-dp) and transformed into other products, such as performance infills for artificial turfs and playground safety floorings [28,29,30]. However, regarding the potential toxicity of these substances, few studies (some of which are very old) have been conducted, and many research gaps currently affect the available knowledge, such as the lack of data on long-term toxicity; the differences in the results expression; and terminological inconsistency about TWP, TRWP and ELT [10,14]. For instance, a study on the crustacean *Hyallela azteca* revealed acute and chronic effects of TWPs after an exposure for 21 days to 500–2000 particles/mL as significant impacts on mortality, reproduction and growth [31], identifying 50% lethal concentration (LC_50_) at 3426 ± 172 particles/mL. Another study revealed the absence of effects of ELTs at 10% sediment dry weight after 28 days of exposure in the freshwater crustaceans *Gammarus pulex* and *Asellus aquaticus* and in the Oligochaeta worms *Tubifex* spp. [32]. On the other hand, a larger number of studies have reported the toxicity of tire particle leachates [10,14]. A study on the rainbow trout *Oncorhynchus mykiss*, the crustacean *Daphnia magna* and the fathead minnow *Pimephales promelas* exposed to a leachate of worn, pristine and breakwater tires revealed no effects on *D. magna* and *P. promelas* and a 96 h LC_50_ for *O. mykiss* at 11.8–19.3% *v/v* for old tire leachate, which is more toxic than leachate from new tires [33]. On *O. mykiss*, an increase in ethoxyresorufin-O-deethylase (EROD) activity after exposure to water with whole tires was also observed [34]. Other studies highlighted a wide range of 50% effect concentration (EC_50_) on *D. magna* after 48 h exposure to TWP leachates, ranging from 0.13 to 10 g/L [35,36], and 0.55 to 5 g/L for *Ceriodaphnia dubia* [37]. On the contrary, no significant effects were obtained in *H. azteca*, *P. promelas*, *C. dubia* and in the Diptera *Chironomus dilutus* exposed for 42 days to TRWP leachate (10 g/kg sediment) [38]. The effects of TRWPs on *Pseudokirchneriella subcapita*, *D. magna* and *P. promelas* were also evaluated using sediment elutriate and under standard test temperatures, reporting an EC_50_ greater than 10,000 mg/L [39]. Lastly, in another study, the toxicity of micro- (1–20 µm) and nano- (<1 µm) tire particles (from 0 to 3.0 × 10^9^ particles/mL), as well as their leachate (from 0 to 100%), was assessed on *Danio rerio* and *D. magna*. This study, which combined the assessment of both particles and leachate toxicity, reported mortality and malformations on exposed specimens, especially those treated with nanoparticles [40].

Based on these complicated and heterogeneous pieces of evidence and considering the sparse information about the potential effects related to ELTs in particular, we evaluated the acute and chronic effects induced by aqueous suspensions of ELT-dg (size ranging from 0.8 to 2.5 mm) and ELT-dp (size < 0.8 mm) on freshwater organisms. The material used in the ecotoxicity tests were derived from the mechanical shredding of whole tires containing all compounds used in tire production (inner liners, treads, etc.), as well as the substances normally detected in TRWPs, such as bituminous residues and agglomerates of exhaust fumes. We tested different ELT-dg and ELT-dp concentrations, from 0.12 to 100.0 mg/L, using a battery of classical ecotoxicological tests on freshwater species, such as the unicellular green alga *P. subcapitata*, the crustacean *D. magna* and the teleost *D. rerio* (zebrafish), which constitute a simple aquatic trophic chain.

## 2. Materials and Methods

### 2.1. ELT-dg and ELT-dp Collection

Sampling of ELT-dg was conducted during July 2018 for one week in 20 different ELT treatment plants (19 in Italy and 1 in Switzerland) involved in the Ente Nazionale Italiano di Unificazione (UNI) project of GL14 group (ConfoReach-GVG group). Sampling was performed before the bagging step on particles ranging from 0.8 to 2.5 mm in size using a graduated jag to collect 400 g of selected material for each of the 5 samplings/day repeated for 5 days in a week (25 different samples). At the end of each sampling day, the collected materials were pooled and stored in glass bottles, obtaining 20 different samples (one for each selected plant) [41,42,43,44,45]. Samples were stored avoiding exposure to heat sources and UV rays. To reduce the amount of material, the ELT-dg samples were pooled and reduced to a primary sample of 2 kg by successive stages of splitting. Subsequently, 2 secondary samples of 1 kg each were obtained; one of them was used for ELT characterization, whereas the other one was used for ecotoxicity tests. To obtain the ELT-dp, a fraction of ELT-dg sample was subjected to gridding (cracker mill machine; mesh of 0.8 mm) to reach a powder size < 0.8 mm for ecotoxicity evaluation.

### 2.2. ELT Characterization and Preparation of Aqueous Suspensions

In the 20 different solid samples of ELT-dg, 152 chemicals were preliminarily quantified (Table S1, see references to the methods followed for the determination of each chemical). In addition, the ultrastructure of both ELT-dg and ELT-dp was investigated using scanning electron microscopy (SEM). Selected materials were placed on aluminum stubs with a carbon tape, gold-coated and observed with an FE-SEM SIGMA (Zeiss, Oberkochen, Germany) operating at 5 kV with a working distance of 20 mm [46].

According to the concentrations of chemicals detected in the ELT-dg (Table S1) and considering the theoretical ecotoxicity of these substances/elements as identified by the European Union CLP (classification, labelling and packaging) Regulation (e.g., hazard statements H400, H410, H411, H412 and H413), we selected 20 chemicals (Table S2) with a potential ecotoxicological implication. First, to evaluate the effective detection of these substances/elements in the exposure media from ELT-dg suspension, we calculated their theoretical concentrations in a water fraction of 100.0 mg/L ELT-dg. This concentration used for the ecotoxicity tests was chosen according to the Organization for Economic Co-operation and Development (OECD) guidelines [47,48,49,50]. According to the theoretical results (Table S2), the majority of the 20 selected chemicals were not detectable in water (concentrations < 1 µg/L), except for Zn, Cobalt (Co), Copper (Cu), 2-mercaptobenzothiazole, 4-t-octylphenol, aniline and N-cyclohexyl-cyclohexanamine. To confirm this aspect experimentally, we prepared a suspension of 100.0 mg/L ELT-dp from the 20 pooled samples of ELT-dg in ultrapure water, mixing for 48 h and filtering through 0.45 µm filters. The ELT-dp was chosen, assuming that its homogenization was better than that of ELT-dg due to the smaller size of particles. Subsequently, we quantified the selected 20 chemicals in the water fraction (Table S2, see references to the methods followed for the determination of each chemical). The analytical results showed that Zn was the most abundant solubilized component (53.4 µg/L; coefficient of variation or relative standard deviation, RSD = 2.0%; Table S2). For this reason, Zn was chosen as the main solubilized and quantified component among the 20 selected chemicals with a possible ecotoxicological effect during the exposure.

The suspensions of ELT-dg and ELT-dp were prepared by mixing these materials in water and settling to separate the coarse components [51]. In particular, to identify the suitable mixing time, two suspensions of 100.0 mg/L of both ELT-dg and ELT-dp were mixed for 144 h in ultrapure water, and the concentration of Zn was assessed at 24, 48, 72 and 144 h. After 72 h of mixing, we obtained 80% Zn solubilization and decided to use this time to mix the suspensions for the preparation of test media. Regarding the settling time, two other suspensions of 100.0 mg/L of both ELT-dg and ELT-dp were mixed for 24 h and decanted for 144 h, and the total organic carbon (TOC; <3 mg/L, determination by cuvette test LCK 385, Hach), UV-VIS Spectrum (no significant absorption was observed in the UV-vis spectrum) and Zn concentrations were assessed. The quantification of Zn showed a stable concentration of this element after 48 h of settling, and this time was used for the suspension sedimentation. Lastly, considering that the majority of coarse ELT-dg and ELT-dp floated on the water surface and only a few amount of debris decanted, we prepared the tested media by serial dilutions of 100.0 mg/L, collecting the water from the center of suspensions using a peristaltic pump, avoiding the collection of coarse ELT-dg and ELT-dp from the surface or the bottom of used containers.

### 2.3. Characterization of ELT Suspensions

Because each model organism used in this study has a different exposure medium, we prepared the ELT suspensions separately for each exposure test. Zn concentration was measured during the entire exposures on one sample obtained on water mix from the different exposure tanks. For this reason, the standard deviation for these data was not calculated, and only the RSD, provided by instrumentation on 3 technical measurements, was reported. In detail, water samples were taken from the exposure tanks and acidified with 0.5% *v/v* nitric acid (HNO_3_), stored at room temperature in dark conditions and analyzed within 7 days. The analyses of Zn were performed using a plasma optical emission spectrometer (ICP/OES; Agilent model 5110). Zn in a multi-element standard solution at 100.0 mg/L was used as reference material to prepare diluted standard solutions. In addition, to evaluate the performance of the method, two aliquots of 40 mL water medium were spiked with Zn concentrations from 1.5 to 80 µg/L. The limit of quantification of the analytical method depended on the basal level of Zn in aqueous media and was 1.5 µg/L in both fish and *D. magna* acute test media, 2.3 µg/L in the algal medium and 13.0 µg/L in the *D. magna* reproduction medium (see the Supplementary Materials for medium composition).

To investigate the presence and size of ELT particles in the test media, we performed both SEM and dynamic light scattering (DLS) analyses. In detail, SEM analysis was applied to 100.0 mg/L of ELT-dg and ELT-dp suspensions, sampling the aliquots from the water column and avoiding the collection of floating or decanted coarse particles. Samples were placed on aluminum stubs with a carbon tape, gold-coated and observed with an FE-SEM SIGMA (Zeiss, Oberkochen, Germany) operating at 5 kV with a working distance of 20 mm [46]. Once the presence of particles in the 100 mg/L suspensions was ascertained, we also investigated this aspect with DLS measurements, using a Malvern Zetasizer Nano ZS instrument (Malvern Instruments Ltd., Malvern, UK) equipped with a solid-state He-Ne laser with a wavelength of 633 nm, collecting the scattered light at 173°. The measurements on ELT suspensions were repeated 3 times using a disposable cuvette, leaving 30 s for temperature equilibration. The correlograms were fitted by a *non*-negative least square regression using multiexponential decay with Zetasizer Nano Series Software 7.02.

### 2.4. Toxicity Studies

The concentrations of ELT suspensions tested in the acute assays were chosen based on both OECD guidelines and on results of the range-finding tests (performed at 1.0, 10.0 and 100.0 mg/L of ELT-dg and ELT-dp), in which we did not observe significant effects. For this reason, as defined by OECD guidelines, we performed the limit tests only at 100.0 mg/L. For the subsequent chronic tests, the concentrations were determined in accordance with the results of acute tests. The ecotoxicity methods followed in this study are described in detail in the OECD guidelines [47,48,49,50,52], and for this reason we report only a brief description of them below.

#### 2.4.1. Growth Inhibition Test on *P. subcapitata*

A chronic toxicity test was conducted on *P. subcapitata* with 72 h exposure under static conditions to 1.0, 3.1, 9.8, 31.3 and 100.0 mg/L of both ELT-dg and ELT-dp suspensions [47]. We evaluated the percentage of cell growth rate and yield inhibition at 24, 48 and 72 h. The organisms were cultured in the facility of ChemService Controlli e Ricerche s.r.l. in a climatic chamber at 24 ± 2 °C, 4440–8880 lx in the spectral range of 400–700 nm and maintained under continuous shaking to guarantee both the cell suspension and the correct concentration of carbon dioxide (CO_2_). Only the algal cultures showing an exponential growth were used in the exposure, and the procedures were conducted under a laminar flow hood to avoid algal contamination. For the exposure, 100 mL capacity conical glass flasks with algal medium (see Supplementary Materials for composition) were used, with 3 replicates for each concentration and 6 replicates for the control. In each flask, 50 mL of test solution was poured and inoculated with the algae (10^4^ cells/mL). Algal density was measured every 24 h by diluting an aliquot in 9 g/L sodium chloride (NaCl) solution and reading the algal cell amount with a cell counter (Beckman coulter Z2).

#### 2.4.2. Acute and Chronic Tests on *D. magna*

The acute toxicity test on *D. magna* for both ELT-dg and ELT-dp suspensions was conducted in a 48 h exposure under static conditions to 100.0 mg/L [48]. In particular, we evaluated the immobilization, defined as organisms not able to swim within 15 s after gentle agitation. Specimens (daphnid age < 24 h) obtained by ChemService Controlli e Ricerche s.r.l. breeding were exposed in 40 mL beakers with pre-aerated (oxygenation > 5.62 mg/L) reconstituted water (see Supplementary Materials for composition), with 4 replicates *per* treatment (5 daphnids *per* beaker) at 20 °C, with 16 h of light and 8 h of darkness (1188–1401 lx). The immobilization was checked after 24 and 48 h. The chronic test on *D. magna* for both ELT-dg and ELT-dp was conducted in 21 days of exposure in semi-static condition (renewal 3 times *per* week) to 1.0, 3.1, 9.8, 31.3 and 100.0 mg/L [50]. In this context, after 21 days of exposure, we evaluated the mortality rate and reproduction parameters (total number of living offspring). Specimens (daphnid age < 24 h) were exposed in 50 mL beakers with pre-aerated (oxygenation > 5.00 mg/L) reconstituted water (see the Supplementary Materials for the composition) with 10 replicates *per* treatment (10 daphnids *per* treatment, 1 specimen *per* beaker) at 20 °C, with 16 h of light and 8 h of darkness (1236–1467 lx). During exposure, daphnids were feed with *P. subcapitata* after the water change.

#### 2.4.3. Acute and Chronic Tests on *D. rerio*

The acute toxicity test on *D. rerio* for both ELT-dg and ELT-dp suspensions comprised a 96 h exposure to 100.0 mg/L under static conditions [49]. Fish were considered dead if no movements were visible and if no reaction was observable when touching the caudal peduncle; in addition, we checked for visible abnormalities after 2, 24, 48, 72 and 96 h. Juvenile fish were obtained by ChemService Controlli e Ricerche s.r.l. facility and fed up to 24 h before the test. The facilities of ChemService Controlli e Ricerche s.r.l. follow Italian laws, rules and regulations (Legislative Decree No. 116/92; authorization n. 30/2012-A of 25 January 2012). Fish sizes were checked before the exposure by measuring a representative group of fish of the same batch. For ELT-dg exposure, mean length was 2.40 ± 0.26 cm and mean weight was 0.25 ± 0.04 g, whereas for ELT-dp exposure, mean length was 1.80 ± 0.17 cm and mean weight was 0.30 ± 0.04 g. Organisms of 2 months were exposed in 60 L glass aquaria (7 specimens *per* treatment) filled with reconstituted water (see Supplementary Materials for composition), with one replicate *per* concentration at a temperature of 22 °C, with 12 h light (680–827 lx) and 12 h darkness daily photoperiod (2 daily 30 min transition periods; 754–827 lx), oxygenation > 60% and 6.60–7.77 pH range. The chronic toxicity test on *D. rerio*, for both ELT-dg and ELT-dp suspensions, comprised 30 days of exposure to 0.12, 0.37, 1.1, 3.3 and 10.0 mg/L under semi-static conditions, renewing the media 3 times *per* week [52]. The concentrations were chosen according to OECD 210 [52], and the highest concentration was adopted in accordance with both acute (LC_50_ > 100.0 mg/L) and fish embryo tests (FET; conducted as previous screening; no mortality was observed at 100.0 mg/L). During the test, we evaluated different end points, e.g., hatching, survival, abnormal behavior, weight and length of juvenile fish. For the embryo stage, fish were exposed in glass containers with 50 mL ELT-dg and ELT-dp suspensions, whereas for the juvenile stage, the volume was increased up to 1200 mL. Four replicates *per* treatment (20 eggs *per* well) were used. The exposure was conducted at a temperature between 25.1 and 26.0 °C, with 12 h light (602–853 lx) and 12 h darkness daily photoperiod, oxygenation > 60% and 7.75–7.88 pH range. Fish were fed *ad libitum* starting from 2 days after hatching (the amount was incremented during the test period; Gemma Micro 75, Skretting for larvae and Gemma Micro 150 for juveniles).

### 2.5. Statistical Analyses and Data Elaboration

To avoid confusion between the specific end points evaluated for each selected ecotoxicological test, we summarized and reported the main results as EC_50_, no observed effect concentration (NOEC) and lowest observed effect concentration (LOEC). The variance and distribution of data were verified by Bartlett and Shapiro–Wilk tests. For the growth inhibition test on *P. subcapitata*, the EC_50_ was determined by linear interpolation method for growth rate and by *non*-linear regression analysis for yield. The NOEC and LOEC for growth rate and yield were determined by Bonferroni correction test. For acute and chronic tests on *D. magna*, the EC_50_ was determined with linear interpolation, and NOEC and LOEC were determined by the Steel many-one rank sum test. For acute and chronic tests on *D. rerio*, the EC_50_ was measured by linear interpolation, and NOEC and LOEC were calculated, where possible, by Fisher exact test and Dunnett’s multiple comparison test. CETIS v.1.8.7.7 software was used to carry out these analyses.

In addition, as further data elaboration, we performed a comparison between treated and control, time *versus* time (where possible) of selected end points. For this purpose, we used one/two-way analysis of variance (ANOVA) or a *non*-parametric test if normality or homoscedasticity were not verified, followed by Bonferroni correction test. STATISTICA 7.1 Software was used in these analyses.

## 3. Results and Discussion

### 3.1. Zn and ELT Particle Detection in the Exposure Media

**Figure 1:**
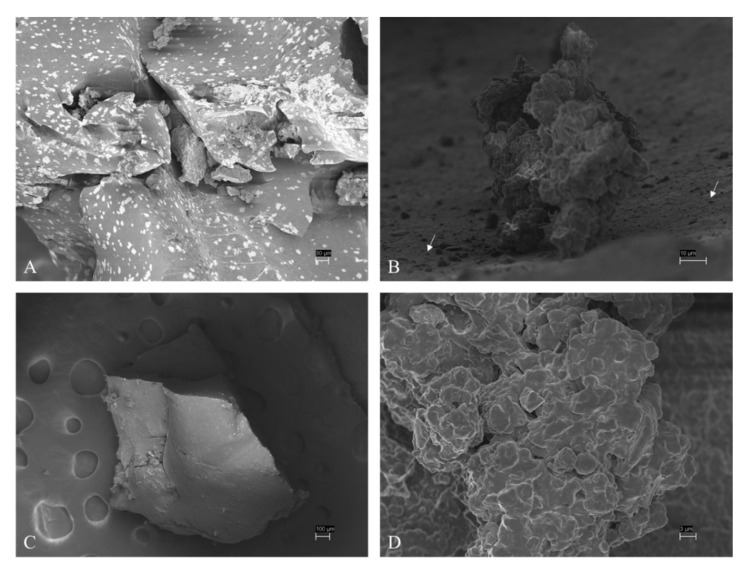
SEM analysis of both ELT-dg (**A**,**B**) and ELT-dp (**C**,**D**). There is wide range of sizes and shapes of selected materials, with nanoparticles (size < 1 µm, based on classification proposed by Hartmann et al. [6] on plastic size) on the surface of a single ELT-dg (**B**) debris (indicated by arrows).

**Figure 2:**
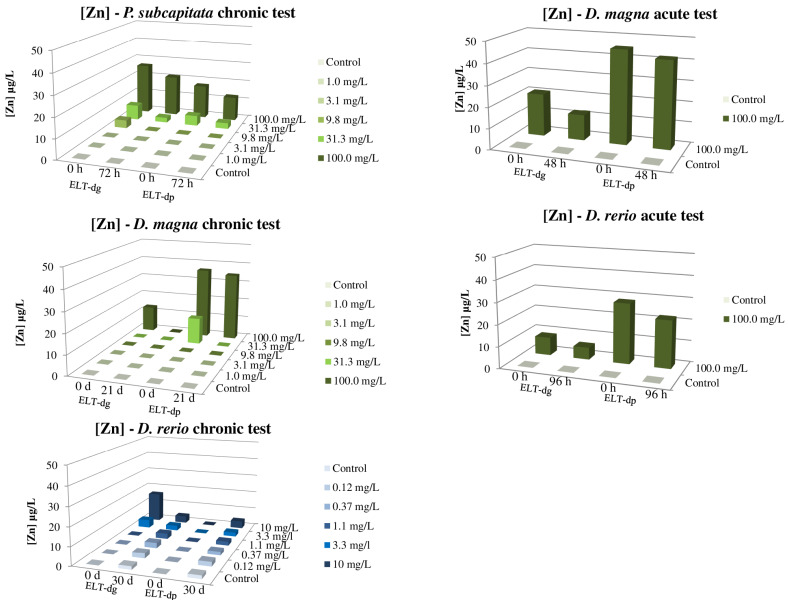
Zn *versus* ELT-dg and ELT-dp concentrations in the suspensions used in the different ecotoxicological tests at the beginning (t = 0) and at the end of each exposure. The concentration of Zn at t = 0 (fresh suspension) corresponds to Zn directly released by ELT-dg and ELT-dp in water (RSD range for groups of *P. subcapitata* = 0.22–1.43%; RSD range for groups of *D. magna* = 0.16–2.91%; RSD range for groups of *D. rerio* = 0.76–5.97%).

SEM analysis of ELT-dg and ELT-dp revealed a heterogeneous size of selected particles, with a complex ultrastructure represented by a wide plethora of rubber shapes (Figure 1). This aspect could be extremely important for understanding the releasing behavior of Zn by selected materials in the aqueous suspensions. In this context, the presence of Zn in ELTs is associated with its use as an activator in the vulcanization process [53], making this element an environmental marker of tire particles [10]. For this reason, the monitoring of Zn in the aqueous suspensions used in the present study could be pivotal for the interpretation of the ecotoxicity results. Regarding the chronic test on *P. subcapitata*, Zn concentrations at the beginning of the test were under the detection limit for the lowest concentrations of ELT-dg (1.0 and 3.1 mg/L) and of 4.1, 7.7 and 26.3 µg/L for ELT-dg concentrations of 9.8, 31.3 and 100.0 mg/L, respectively (Figure 2). At the end of the static exposure (72 h), the concentration of Zn related to the 9.8 mg/L of ELT-dg decreased under the detection limit and to 2.5 and 21.0 µg/L for the highest ELT-dg concentrations of 31.3 and 100.0 mg/L. For the ELT-dp at the beginning of *P. subcapitata* exposure, Zn concentrations were under the limits of detection/quantification in the groups from 1.0 to 9.8 mg/L of ELT-dp and 5.2 and 17.3 µg/L for the highest concentrations of 31.3 and 100.0 mg/L. Similarly, at the end of the exposure, these values remained under the detection/quantification limits for concentrations up to 9.8 mg/L, whereas we obtained values of 3.0 and 12.4 µg/L for 31.3 and 100.0 mg/L, respectively. Regarding the acute test on *D. magna*, the concentration of Zn in the aqueous suspension from 100.0 mg/L of ELT-dg at the beginning of the static test was 20.7 µg/L, decreasing to 12.3 µg/L at the end of exposure (48 h). On the contrary, for 100.0 mg/L of ELT-dp, we obtained the highest and most constant concentrations during the static exposure, with 45.1 µg/L at time 0 and 41.8 µg/L at the end of the test. Similarly, in the chronic test on *D. magna* under semi-static conditions, Zn concentration for the ELT-dg suspension was measured only in the highest tested concentration of 100.0 mg/L because preliminary analytical tests showed that at the lower tested concentrations, Zn was not quantifiable. During the 21 days of exposure, the concentration of Zn detected was between 14.6 µg/L (maximum measured concentration; MaMC) and 5.0 µg/L (minimum measured concentration; MiMC). Regarding the ELT-dp suspension, Zn concentration was measured, once again, only in the two highest tested concentrations of 31.3 and 100.0 mg/L. During the 21 days of exposure, the Zn concentration detected in 31.3 mg/L suspension was between 17.2 µg/L (MaMC) and 13.0 µg/L (MiMC), whereas the concentration of Zn detected in 100.0 mg/L was between 49.5 µg/L (MaMC) and 22.9 µg/L (MiMC). Due to the large amount of data, in Figure 2, we reported only the concentrations of Zn at the beginning and at the end of each exposure, but it is important to note that in the chronic tests, the media were renewed every 3 days, and every 3 days, the concentrations of Zn in fresh and spent solutions were measured. Therefore, for chronic tests, the concentrations of Zn at the beginning and at the end of the tests are not directly related. Moving to the acute test on *D. rerio*, the concentration of Zn in the suspension of 100.0 mg/L of ELT-dg was 8.4 µg/L at the beginning of static-exposure, with a slight reduction to 5.6 µg/L at the end of the test (96 h). Similarly, we obtained a decrease in Zn concentration in the suspension from ELT-dp, where the concentration was 28.1 µg/L at the beginning of the test and then reduced to 22.2 µg/L at the end of the exposure. Lastly, in the chronic test on *D. rerio* under semi-static conditions, for ELT-dg suspension of 10.0 mg/L, the concentration of Zn detected was between 16.3 µg/L (MaMC) and 1.5 µg/L (MiMC); for ELT-dg suspension of 3.3 mg/L, the concentration of Zn detected was between 9.4 µg/L (MaMC) and 0.5 µg/L (MiMC). For ELT-dp suspension of 10.0 mg/L, the concentration of Zn detected was between 11.0 µg/L (MaMC) and 0.5 µg/L (MiMC); for ELT-dp suspension of 3.3 mg/L, the concentration of Zn detected was between 6.2 µg/L (MaMC) and 0.5µg/L (MiMC). In this context, we also detected Zn in the control at the end of exposures (Figure 2). According to preliminary analytical stability tests, it can be stated that Zn is stable for 3 days; when the food supply started, Zn was influenced by the presence of dissolved food and/or uneaten food and feces.

In general, Zn began to be released in water at appreciable concentrations starting from 10.0 and 31.3 mg/L of ELT-dg and ELT-dp. A similar trend in the release of this element in water was observed at a concentration of 100.0 mg/L for both ELT-dg and ELT-dp, as represented by the histograms at t = 0 h (fresh solution; Figure 2). This aspect suggests that the smaller size of ELT-dp caused a higher release of Zn, probably due to the increase in the surface/volume ratio of particles. Indeed, despite it has been demonstrated that the leaching of some elements from crumb rubbers was size-independent [8], in the present study the release of Zn was higher by ELT-dp, as observed in the tests on *D. magna* and *D. rerio* (Figure 2). In this context, some evidence highlighted that the leaching of Zn from TWPs was reduced by an increase in salinity and pH and enhanced by fluorescent light compared to dark conditions [54]. However, because an unclear trend related to salinity or pH of water media was observed in our work, further investigations are needed to clarify the role of chemical/physical parameters in the release of Zn by both ELT-dg and ELT-dp.

In the context of Zn release, Capolupo et al. [55] reported that this element was the main chemical detected in car tire rubber leachate, together with benzothiazole and Co. The fluctuating values of Zn obtained at the beginning of each exposure were probably related to the heterogeneity of sizes and shapes of ELT particles (Figure 1). This aspect, which cannot be controlled during the preparation of aqueous suspensions (each weighing of ELT material contains very different particles), could cause a heterogeneous Zn release in water in a size/shape-dependent manner.

**Figure 3:**
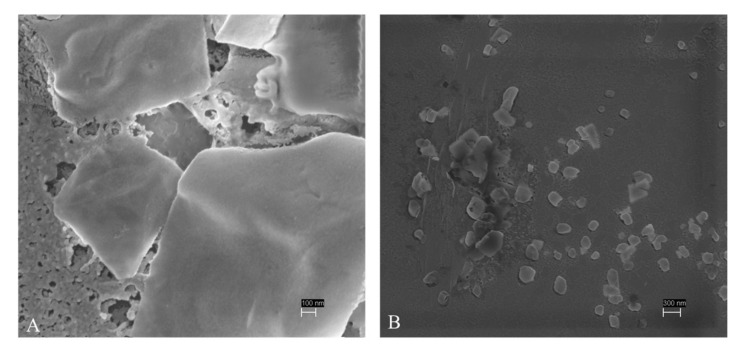
SEM analysis of both ELT-dg (**A**) and ELT-dp (**B**) aqueous suspensions of 100.0 mg/L. These images confirm the presence of some micro- and nanoparticles (size < 1 µm, based on classification proposed by Hartmann et al. [6] on plastic size) in the selected exposure media.

The presence of ELT particles was certified in some ELT suspensions through SEM and DLS analyses. At the highest concentration of 100.0 mg/L, we detected the presence of both ELT-dg and ELT-dp nanoparticles (size < 1 µm, based on classification proposed by Hartmann et al. [6] on plastic size; Figure 3) in the aqueous fraction. Consequently, we investigated this aspect with DLS in the other ELT suspensions. Concerning ELT-dg, we observed particles with a mean size of 810 ± 215 nm, 1290 ± 520 nm and 1508 ± 615 nm in the suspensions of 100.0, 31.3 and 10.0 mg/L, respectively. This evidence highlighted the presence of both nano- and microparticles in ELT-dg suspensions. For the other suspensions, no repeatable data were obtained, probably due to the serial dilutions, which decreased the number of particles under the detection limit of DLS instrumentation. Concerning ELT-dp, readings appeared more unstable than for ELT-dg, resulting in a change in the correlation function. We observed a value of 530 ± 109 nm in the 100 mg/L suspension. In the other dilution of 31.3 mg/L, correlograms were close to the detection limit, with two particle populations identified, one of 478 ± 106 nm and another one exceeding the range covered by the DLS instrument of about 8000 nm.

### 3.2. Acute and Chronic Effects of ELT-dg and ELT-dp Suspensions

Regarding the acute test on *D. magna*, we did not observe immobilization in the control group, and no daphnids were trapped on the water surface during the exposure. In addition, daphnids did not show signs of disease or stress in the controls during the test. We obtained a value of 24 and 48 h EC_50_ for ELT-dg and ELT-dp suspensions > 100.0 mg/L (Table 1). Based on Zn concentrations measured in the exposure media at the beginning and at the end of exposure, we performed a time-weighted arithmetic mean of Zn concentrations between time 0 and 48 h [50], obtaining an EC_50_ > 16.2 µg/L of Zn for ELT-dg and an EC_50_ > 43.5 µg/L of Zn for ELT-dp at both 24 and 48 h of exposure. In the same manner, no mortality or anomalies were observed in *D. rerio* in both control and treated groups. On *D. rerio*, we obtained an EC_50_ > 100.0 mg/L for both ELT-dg and ELT-dp suspensions from 24 to 96 h of exposure. Regarding the concentration of Zn, we obtained an EC_50_ > 6.9 µg/L of Zn for ELT-dg and an EC_50_ > 25.0 µg/L of Zn for ELT-dp suspension (see also the Zn concentrations in the water media reported in Figure 2).

**Figure 4:**
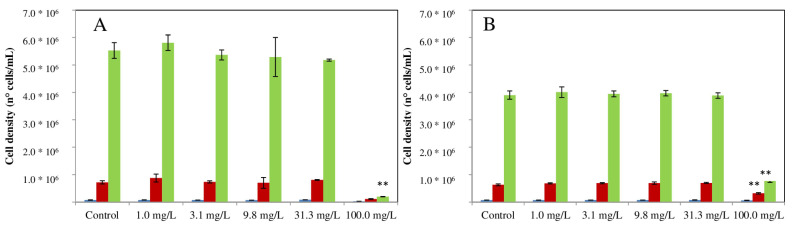
Significant effects indicated by asterisks (** *p* < 0.01), control *versus* treated, induced by both ELT-dg (**A**) and ELT-dp (**B**) suspensions in *P. subcapitata* (cell density) at 24 (blue bars), 48 (red bars) and 72 h (green bars).

Regarding the test on the green alga *P. subcapitata*, for both ELT-dg and ELT-dp, we obtained a LOEC of 100.0 mg/L from 0 to 72 h. In this context, as reported in Figure 4, we observed a significant effect of treatment on the cell density (we used the cell density to calculate the growth rate and yield) of *P. subcapitata* exposed to ELT-dg at the end of exposure to 100.0 mg/L (*p* < 0.01; Figure 4A) and in algae exposed to 100.0 mg/L of ELT-dp at 48 h (*p* < 0.01) and 72 h (*p* < 0.01; Figure 4B).

**Figure 5:**
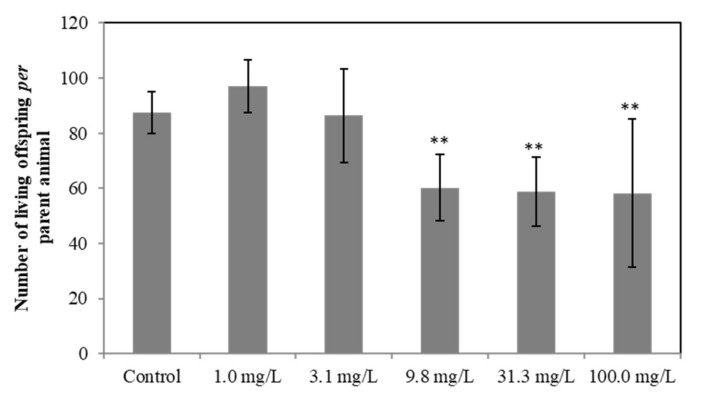
Significant effects indicated by asterisks (** *p* < 0.01), control *versus* treated, induced by ELT-dg suspension in *D. magna* (number of living offspring).

At the higher trophic level, in the chronic test on *D. magna*, we observed a value of 21 days LOEC in the exposure to the suspension from ELT-dg, referring to the reproduction, of 9.8 mg/L. In this context, we obtained the following total number of living offspring produced *per* parental animal: 87.4 ± 7.6 in the control, 97.1 ± 9.6 at 1.0 mg/L, 86.4 ± 17.0 at 3.1 mg/L, 60.2 ± 12.0 at 9.8 mg/L, 58.8 ± 12.5 at 31.3 mg/L and 58.2 ± 26.9 at 100 mg/L. Coherently with the LOEC, we observed a significant effect of treatment on the number of living offspring, with a significant reduction compared to the control (*p* < 0.01) at the three highest concentrations (9.8, 31.3 and 100.0 mg/L; Figure 5). Conversely, for all other considered parameters (mean number of dead offspring, aborted eggs and body length of parent animals), no significant effects were observed (data not shown). Regarding the ELT-dp, we did not observe any significant effect compared to the control, whereas we obtained a LOEC > 34.5 µg/L of Zn. In this context, for *P. subcapitata*, *D. magna* and *D. rerio* chronic tests, toxicity results expressed as mean Zn concentrations (time-weighted arithmetic mean, measured at the beginning and at the end of the test) were not determined because it was not possible to quantify their concentration for some samples, as reported in Section 3.1.

**Figure 6:**
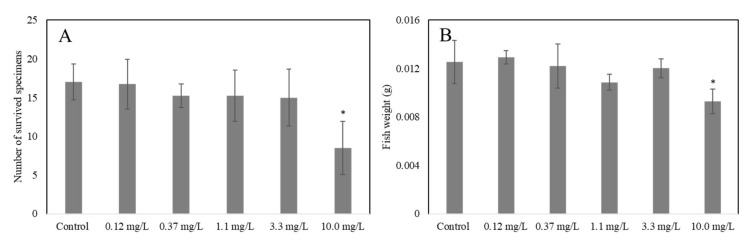
Significant effects indicated by asterisks (* *p* < 0.05), control *versus* treated, induced in *D. rerio* by both ELT-dp ((**A**); survival) and ELT-dg ((**B**); fish weight) suspensions.

In *D. rerio*, for ELT-dg and ELT-dp suspensions, the test started at 4–8 cells, corresponding to 1.25 hpf. In both tests, we observed hatching of 100% (LOEC > 10.0 mg/L; Table 1). For the survival parameter, we obtained a LOEC of 10.0 mg/L on the specimens exposed to ELT-dg and ELT-dp. In addition, for the juvenile fish weight parameter, the LOEC for specimens exposed to ELT-dg was 10.0 mg/L, as well as for the abnormal behavior, only in fish exposed to ELT-dp (Table 1). In this context, we observed a significant reduction in survival (*p* < 0.05) and fish weight (*p* < 0.05) in *D. rerio* exposed to 10.0 mg/L ELT-dp and ELT-dg suspensions, respectively (Figure 6A,B).

In some studies, leachates from tires generally show low toxicity on aquatic species, with high values of EC_50_ between 0.1 g/L and 100 g/kg [56]. As observed in the present study, the toxicity of ELT suspensions might be associated with the release of metals, such as Zn [56], as well as ELT nano- and microparticles detected in the exposure media. In this context, in future studies, it will also be important to consider the role played by other chemicals released by ELTs, such as 4-(dimethylbutylamino)diphenylamine. In the aquatic environment, this nitrosamine used as an antioxidant in tires produces a very toxic quinone able to induce acute effects on aquatic species at concentrations raging from <0.3 to 19 µg/L, as recently observed in the salmonid *Oncorhynchus kisutch* (LC_50_ 0.8 ± 0.16 µg/L) [57]. However, coherently with our work, Marwood et al. [39] investigated the effects of TRWP sediment elutriate from Michelin, Pirelli and Bridgestone tires using a road simulator laboratory, reporting an EC_50_ higher than 10,000 mg/L for *P. subcapitata*, *D. magna* and *P. promelas*. In this work, Zn and aniline were identified as the main toxic chemicals [39]. The study of Halsband et al. [58] highlighted how crumb rubber granules from ELTs, used as performance infill in synthetic turf pitches as well as their leaching, could pose a potential threat for wildlife, reporting acute effects of leachates within 24 h at the highest tested concentrations (100 and 50 g/L), with 48 h LC_50_ of 35 g/L for *Calanus* sp. and <5 g/L for *Acartia* sp. Once again, benzothiazole and Zn were the main components of the leachates in this study. In this context, some evidence has suggested that when salinity and pH increase, the leaching of Zn is reduced [54] and leachates decrease their toxicity [59]. This aspect could suggest the key role of Zn in the toxicity modulation of leachates. At the same time, when the leachates are obtained at pH < 7, Zn increases together with toxicity [60]. Considering this evidence and that obtained in our study concerning the trends in the release of Zn in water by ELT-dg and ELT-dp, other investigations on the role of Zn in ELT toxicity are necessary. Indeed, without a mechanistic approach, e.g., by the application of biomarkers, such as metallothioneins, cellular stress and oxidative damage end points, it is difficult to determine whether Zn is the main driver of ELT toxicity. Therefore, the effects observed at the organism and population level in this study on reproduction, survival and growth of exposed specimens represent a starting point for further mechanistic studies on ELT impact.

## 4. Conclusions

Considering the obtained results, neither ELT-dg nor ELT-dp can be classified in the context of CLP regulation, either for short-term aquatic hazard (because we observed an EC_50_ > 1 mg/L in acute toxicity tests) or for long-term aquatic hazard (because we observed NOEC > 1 mg/L in chronic tests) for all trophic levels.

The obtained results suggested that ELT suspensions exhibit a threshold of toxicity of 9.8 mg/L for the tested end point at the organism and population level. In this context, future studies on ELTs should focus on the chronic toxicity of these contaminants, as well as characterizing their possible infiltration in the biota tissues. Currently, there is little information in the scientific literature about the ecotoxicological implication of ELTs. This also affects the comparative evaluations between different works and experimental approaches. For this reason, other investigations are urgently needed using, e.g., more sensitive methodologies, such as biomarkers or “omics” techniques, to evaluate ELT effects at the biochemical, molecular and cellular level, delineating their potential mechanism of action. Therefore, the characterization of the ecological impact of ELTs needs more explanations, and this study represents a preliminary work to fill the gap concerning the impact of these materials in freshwater ecosystems.

## Figures and Tables

**Table 1:** Values of EC_50_, NOEC and LOEC for the main tested end points obtained by the different OECD tests for both ELT-dg and ELT-dp suspensions. For EC_50_ determination, the following conditions were evaluated: the 95% confidence interval did not contain zero and was not overly wide, the 95% confidence interval for the predicted mean did not contain the control mean, there was no significant lack of fit of regression model to the data. If the above conditions were not satisfied, the NOEC approach was used.

		Suspension from ELT-dg			Suspension from ELT-dp		
		EC_50_	NOEC (mg/L)	LOEC (mg/L)	EC_50_	NOEC (mg/L)	LOEC (mg/L)
*P. subcapitata* chronic test	Growth rate	93.7 (0–72 h)	31.3 (0–72 h)	100.0 (0–72 h)	>100.0 (0–72 h)	31.3 (0–72 h)	100.0 (0–72 h)
	Yeld	54.2 (0–72 h)	31.3 (0–72 h)	100.0 (0–72 h)	73.6 (0–72 h)	31.3 (0–72 h)	100.0 (0–72 h)
*D. magna* acute test	Immobilization	>100.0 (24 and 48 h)	100.0 (24 and 48 h)		>100.0 (24 and 48 h)	100.0 (24 and 48 h)	
*D. rerio* acute test	Mortality	>100.0 (24, 48, 72 and 96 h)	100.0 (24, 48, 72 and 96 h)		>100.0 (24, 48, 72 and 96 h)	100.0 (24, 48, 72 and 96 h)	
*D. magna* chronic test	Reproduction	>100.0 (21 d)	3.1 (21 d)	9.8 (21 d)	>100.0 (21 d)	100.0 (21 d)	>100.0 (21 d)
	Parental mortality		100.0 (21 d)	>100.0 (21 d)		100.0 (21 d)	>100.0 (21 d)
*D. rerio* chronic test	Hatching		10.0 (96 hpf)	>10.0 (96 hpf)		10.0 (96 hpf)	>10.0 (96 hpf)
	Juvenile survival		3.3 (30 d)	10.0 (30 d)		3.3 (30 d)	10.0 (30 d)
	Juvenile weight		3.3 (30 d)	10.0 (30 d)		10.0 (30 d)	>10.0 (30 d)
	Juvenile lenght		10.0 (30 d)	>10.0 (30 d)		10.0 (30 d)	>10.0 (30 d)
	Abnormal behaviour					3.3 (30 d)	10.0 (30 d)

## Data Availability

Not applicable.

## Supplementary Materials

The following supporting information can be downloaded at: https://www.mdpi.com/article/10.3390/toxics10050201/s1, Supplementary Methods; Table S1: Chemicals (mg/Kg) quantified in the 20 different ELT-dg samples (solid fraction). Zinc oxide, Titanium oxide and Magnesium oxide were not detected analytically in the samples and for this reason their presence in the ELT-dg was not certified.; Table S2: Twenty selected chemicals with the concentration detected in the ELT suspensions. In the table are reported the CAS number, the used methods for the chemical detection, the CLP classification as well as the theoretical and analytical concentration of considered substances/elements. In particular, in the column (A) it is reported the max concentration measured in the 100.0 mg/L of ELT-dg suspensions (mg of each component in 1 Kg of ELT-dg, corresponding to µg in 1 g of ELT-dg; Table S1), in the column (B) the µg component in 100.0 mg of sample (calculated form max concentration), in the column (C) the theoretical concentration calculated assuming the hypothesis that all the amount present in 100.0 mg of ELT-dg was dissolved/solubilized in 1 L of water and, lastly, in the column (D) the analytical concentration measured in the 100.0 mg/L of ELT-dp.
