# Supplementary material for: Ecological Impact of End-of-Life-Tire (ELT)-Derived Rubbers: Acute and Chronic Effects at Organism and Population Levels
Source: Toxics. 2022 Apr 19;10(5):201. doi: 10.3390/toxics10050201 (PMC9144162; doi:10.3390/toxics10050201)
Supplement: Supplementary file 1 [file toxics-10-00201-s001.zip › Supplementary Methods.pdf]

Composition of reconstituted water used in the ecotoxicological tests

Acute and chronic tests on zebrafish (OECD 203, 210)
Acute test on *D. magna* (OECD 202)

Reconstituted water: analytical grade salts will be dissolved in purified water (conductivity < 10 µS/cm) to obtain the following nominal concentrations

$CaCl_2 * 2H_2O$	:	2.0 mmol/L (= 294.0 mg/L)
$MgSO_4 * 7H_2O$	:	0.5 mmol/L (= 123.3 mg/L)
$NaHCO_3$	:	0.771 mmol/L (= 64.8 mg/L)
KCl	:	0.078 mmol/L (= 5.8 mg/L)
Water Hardness	:	10 - 250 mg/L as $CaCO_3$
Alkalinity	:	0.8 mmol/L
Ratio of		Ca : Mg = 4 : 1 (based on molarity)
		Na : K = 10 : 1 (based on molarity)

Growth inhibition test on *P. subcapitata* (OECD 201)

Algal medium: in deionized water (conductivity < 5 µS/cm) analytical grade salts will be added to following final nominal concentrations:

Macro-nutrients:	$NaHCO_3$	50.0 mg/L
	$CaCl_2 * 2\ H_2O$	18.0 mg/L
	NH_4Cl	15.0 mg/L
	$MgSO_4 * 7\ H_2O$	15.0 mg/L
	$MgCl_2 * 6\ H_2O$	12.0 mg/L
	KH_2PO_4	1.6 mg/L
Trace Elements:	$Na_2EDTA * 2\ H_2O$	100.0 µg/L
	$FeCl_3 * 6\ H_2O$	64.0 µg/L
	$MnCl_2 * 4\ H_2O$	415.0 µg/L
	H_3BO_3	185.0 µg/L
	$Na_2MoO_4 * 2\ H_2O$	7.0 µg/L
	$ZnCl_2$	3.0 µg/L
	$CoCl_2 * 6\ H_2O$	1.5 µg/L
	$CuCl_2 * 2\ H_2O$	0.01 µg/L

pH 8.1 ± 0.2

Chronic test on *D. magna* (OECD 211)

Reconstituted water:	in deionized water (conductivity < 5 µS/cm), analytical grade, the following salts were added to a defined final nominal concentration:	
Trace Elements:	H_3BO_3	2860 mg/L
	$MnCl_2*4H_2O$	0.361 mg/L
	LiCl	0.306 mg/L
	RbCl	0.071 mg/L
	$SrCl_2*6H_2O$	0.152 mg/L
	NaBr	0.016 mg/L
	$MoNa_2O_4*2H_2O$	0.063 mg/L
	$CuCl_2*2H_2O$	0.017 mg/L
	$ZnCl_2$	0.013 mg/L
	$CoCl_2*6H_2O$	0.010 mg/L
	KI	0.003 mg/L
	Na_2SeO_3	0.002 mg/L
	NH_4VO_3	0.001 mg/L
	$Na_2EDTA*2H_2O$	2.500 mg/L
	$FeSO_4*7H_2O$	0.996 mg/L
Macro-nutrients:	$CaCl_2*2H_2O$	293.8 mg/L
	$MgSO_4*7H_2O$	123.3 mg/L
	KCl	5.8 mg/L
	$NaHCO_3$	64.8 mg/L
	$Na_2SiO_3*9H_2O$	10 mg/L
	$NaNO_3$	0.274 mg/L
	KH_2PO_4	0.143 mg/L
	K_2HPO_4	0.184 mg/L
	Thiamine hydrochloride	75 µg/L
	Cyanocobalamine (B12)	1 µg/L
	Biotine	0.75 µg/L
