# Supplementary material for: Ecological Impact of End-of-Life-Tire (ELT)-Derived Rubbers: Acute and Chronic Effects at Organism and Population Levels
Source: Toxics. 2022 Apr 19;10(5):201. doi: 10.3390/toxics10050201 (PMC9144162; doi:10.3390/toxics10050201)
Supplement: Supplementary file 1 [file toxics-10-00201-s001.zip › Table S1.pdf]

	chemicals	cas	conc.	METHOD	20 different solid samples of ELT-dg																			
					1	2	3	4	5	6	7	8	9	10	11	12	13	14	15	16	17	18	19	20
	Benzo(a)pyrene	50-32-8	mg/kg	ETRMA	1.18	0.72	0.53	0.89	0.73	0.26	0.78	0.72	1.16	0.71	0.66	0.57	0.64	0.6	1.73	1.24	0.62	1.09	0.83	0.78
	Benzo(a)anthracene	56-55-3	mg/kg	ETRMA	1.64	0.88	0.53	0.63	0.4	0.17	0.47	0.5	1.1	0.58	0.7	0.41	0.57	0.18	2.46	1.46	0.38	0.99	0.56	0.42
	Benzo(b)fluoranthene	205-99-2	mg/kg	ETRMA	0.91	0.53	0.32	0.53	0.44	0.13	0.38	0.4	0.86	0.5	0.38	0.3	0.39	0.25	1.62	0.96	0.28	0.69	0.74	0.32
	Benzo(e)pyrene	192-97-2	mg/kg	ETRMA	1.73	1.16	0.77	1.19	1.1	0.51	1.14	1.09	1.56	1.2	0.88	0.82	0.99	0.94	1.81	1.7	0.87	0.74	0.32	< 1
	Benzo(j)fluoranthene	205-82-3	mg/kg	ETRMA	< 1	< 1	< 1	< 1	< 1	< 1	< 1	< 1	< 1	< 1	< 1	< 1	< 1	< 1	< 1	< 1	< 1	< 1	< 1	< 1
	Benzo(k)fluoranthene	207-08-9	mg/kg	ETRMA	< 1	< 1	< 1	< 1	< 1	< 1	< 1	< 1	< 1	< 1	< 1	< 1	< 1	< 1	< 1	< 1	< 1	< 1	< 1	< 1
	Chrysene	218-01-9	mg/kg	ETRMA	1.83	1.1	0.46	0.73	0.63	1.1	1.1	1.1	1.1	1.1	0.85	0.63	0.7	0.51	1.95	1.66	0.45	1.2	1.44	0.72
	Dibenzo(a,h)anthracene	53-70-3	mg/kg	ETRMA	< 1	< 1	< 1	< 1	< 1	< 1	< 1	< 1	< 1	< 1	< 1	< 1	< 1	< 1	< 1	< 1	< 1	< 1	< 1	< 1
	Naphthalene	91-20-3	mg/kg	ETRMA	1	0.86	0.28	0.38	0.43	0.85	1.13	0.49	0.48	0.31	0.24	0.31	0.33	0.4	0.45	0.39	0.38	0.23	0.22	0.37
	Anthracene	120-12-7	mg/kg	ETRMA	1.62	0.98	0.98	0.39	0.29	0.27	0.32	0.27	0.79	0.46	0.41	0.27	0.3	0.26	0.55	0.68	0.31	0.82	0.34	0.29
	Fluoranthene	206-44-0	mg/kg	ETRMA	10.1	7.27	4.18	6.1	5.6	4.9	5.9	6.6	7.6	5.8	3.85	3.97	4.15	4.84	5.38	6.86	6.03	7.09	5.74	5.14
	Dibenzo(a,e)pyrene	192-65-4	mg/kg	ETRMA	< 1	< 1	< 1	< 1	< 1	< 1	< 1	< 1	< 1	< 1	< 1	< 1	< 1	< 1	< 1	< 1	< 1	< 1	< 1	< 1
	Dibenzo(a,h)pyrene	189-64-0	mg/kg	ETRMA	< 1	< 1	< 1	< 1	< 1	< 1	< 1	< 1	< 1	< 1	< 1	< 1	< 1	< 1	< 1	< 1	< 1	< 1	< 1	< 1
	Dibenzo(a,i)pyrene	189-55-9	mg/kg	ETRMA	< 1	< 1	< 1	< 1	< 1	< 1	< 1	< 1	< 1	< 1	< 1	< 1	< 1	< 1	< 1	< 1	< 1	< 1	< 1	< 1
	Anthanthrene	191-26-4	mg/kg	ETRMA	< 1	< 1	< 1	< 1	< 1	< 1	< 1	< 1	< 1	< 1	< 1	< 1	< 1	< 1	< 1	< 1	< 1	< 1	< 1	< 1
PAHs	Acenaphthylene	208-96-8	mg/kg	ETRMA	1.32	1.18	0.45	0.75	1.18	1.98	2.33	0.89	0.9	0.55	0.42	0.5	0.51	0.79	0.72	0.66	0.96	0.5	0.49	0.8
	Indeno(l,2,3-cd)pyrene	193-39-5	mg/kg	ETRMA	0.79	0.46	0.41	0.76	0.6	0.11	0.5	0.48	0.98	0.44	0.59	0.45	0.4	0.43	1.53	1.18	0.53	0.87	0.54	0.71
	Phenanthrene	85-01-8	mg/kg	ETRMA	6.35	3.9	1.53	2.16	1.9	2.2	2.8	2.3	3.6	2.2	1.6	1.59	1.65	1.63	2.11	3.2	1.96	3.01	1.94	1.78
	Acenaphthene	83-32-9	mg/kg	ETRMA	< 1	< 1	< 1	< 1	< 1	< 1	< 1	< 1	< 1	< 1	< 1	< 1	< 1	< 1	< 1	< 1	< 1	< 1	< 1	< 1
	Pyrene	129-00-0	mg/kg	ETRMA	20.7	18.5	14.1	14.2	15	14.2	13.4	17.2	15.3	13.6	8.62	9.0	9.84	13	10.6	15.3	12.8	16.1	12.4	13
	Fluorene	86-73-7	mg/kg	ETRMA	< 1	< 1	< 1	< 1	< 1	< 1	< 1	< 1	< 1	< 1	< 1	< 1	< 1	< 1	< 1	< 1	< 1	< 1	< 1	< 1
	Benzo(g,h,i)perylene	191-24-2	mg/kg	ETRMA	5.19	3.45	3.96	6.78	6	1.13	4.9	4.9	8.1	3.8	4.66	4.16	4.07	4.4	6.97	9.01	4.79	6.8	4.45	6.02
	benzo(c)fluorene	205-12-9	mg/kg	ETRMA	< 5	< 5	< 5	< 5	< 5	< 5	< 5	< 5	< 5	< 5	< 5	< 5	< 5	< 5	< 5	< 5	< 5	< 5	< 5	< 5
	Benzo(b)naphtho(2,1-d)thiophene	239-35-0	mg/kg	ETRMA	< 5	< 5	< 5	< 5	< 5	< 5	< 5	< 5	< 5	< 5	< 5	< 5	< 5	< 5	< 5	< 5	< 5	< 5	< 5	< 5
	Benzo(g,h,i)fluoranthene	203-12-3	mg/kg	ETRMA	2.11	1.78	1.08	1.92	0.73	0.87	0.36	1.98	0.60	1.69	2.19	1.20	1.20	0.73	1.70	4.02	0.71	2.13	2.85	1.33
	Benzo(c)phenanthrene	195-19-7	mg/kg	ETRMA	< 5	< 5	< 5	< 5	< 5	< 5	< 5	< 5	< 5	< 5	< 5	< 5	< 5	< 5	< 5	< 5	< 5	< 5	< 5	< 5
	Coronene	191-07-1	mg/kg	ETRMA	< 5	< 5	< 5	< 5	< 5	< 5	< 5	< 5	< 5	< 5	< 5	< 5	< 5	< 5	< 5	< 5	< 5	< 5	< 5	< 5
	Triphenylene	217-59-4	mg/kg	ETRMA	1.45	1.07	0.44	1.03	0.39	0.47	0.27	1.09	0.40	0.90	1.42	0.65	0.49	0.40	1.00	1.58	0.43	1.18	1.71	0.75
	Perylene	198-55-0	mg/kg	ETRMA	< 1	< 1	< 1	< 1	< 1	< 1	< 1	< 1	< 1	< 1	< 1	< 1	< 1	< 1	< 1	< 1	< 1	< 1	< 1	< 1
	Cyclopenta[cd]pyrene	27208-37-3	mg/kg	ETRMA	< 5	< 5	< 5	< 5	< 5	< 5	< 5	< 5	< 5	< 5	< 5	< 5	< 5	< 5	< 5	< 5	< 5	< 5	< 5	< 5
	9,l0-Dimethyl-1,2-Benzanthracene	57-97-6	mg/kg	ETRMA	< 5	< 5	< 5	< 5	< 5	< 5	< 5	< 5	< 5	< 5	< 5	< 5	< 5	< 5	< 5	< 5	< 5	< 5	< 5	< 5
Metals	Cadmium	7440-43-9	mg/kg	UNI EN 13657:2004 + UNI EN ISO 11885:2009	< 5	< 5	< 5	< 5	< 5	< 5	< 5	< 5	< 5	< 5	< 5	< 5	< 5	< 5	< 5	< 5	< 5	< 5	< 5	< 5
	Mercury	7439-97-6	mg/kg	UNI EN 13657:2004 + UNI EN ISO 11885:2009	< 5	< 5	< 5	< 5	< 5	< 5	< 5	< 5	< 5	< 5	< 5	< 5	< 5	< 5	< 5	< 5	< 5	< 5	< 5	< 5
	Beryllium	7440-41-7	mg/kg	UNI EN 13657:2004 + UNI EN ISO 11885:2009	< 5	< 5	< 5	< 5	< 5	< 5	< 5	< 5	< 5	< 5	< 5	< 5	< 5	< 5	< 5	< 5	< 5	< 5	< 5	< 5
	Lead	7439-92-1	mg/kg	UNI EN 13657:2004 + UNI EN ISO 11885:2009	10.7	8.83	13.9	14.1	14.6	12.9	8.88	12	14.3	14.2	13.3	12.6	10.1	13.3	7.49	9.2	8.53	9.56	11.5	11
	Cobalt	7440-48-4	mg/kg	UNI EN 13657:2004 + UNI EN ISO 11885:2009	110	108	108	112	153	134	124	102	137	109	85.8	102	104	82.8	109	101	114	107	104	110
	Nickel	7440-02-0	mg/kg	UNI EN 13657:2004 + UNI EN ISO 11885:2009	< 5	< 5	< 5	< 5	< 5	< 5	< 5	< 5	< 5	< 5	< 5	< 5	< 5	< 5	< 5	< 5	< 5	< 5	< 5	< 5
	Aluminum	7429-90-5	mg/kg	UNI EN 13657:2004 + UNI EN ISO 11885:2009	78.9	74.8	77.7	73.3	82.8	66.9	80.7	67	103	82.3	79	62.5	97.9	89.5	70.3	76	86	87.6	83.3	76.7
	Zinc	7440-66-6	mg/kg	UNI EN 13657:2004 + UNI EN ISO 11885:2009	9899	9490	10400	9576	10000	10765	10040	10500	9665	10064	9830	9402	9713	10200	9663	9435	10919	9920	9669	9914
	Arsenic	7440-38-2	mg/kg	UNI EN 13657:2004 + UNI EN ISO 11885:2009	< 5	< 5	< 5	< 5	< 5	< 5	< 5	< 5	< 5	5.77	< 5	< 5	< 5	< 5	< 5	< 5	< 5	< 5	< 5	< 5
	Thallium	7440-28-0	mg/kg	UNI EN 13657:2004 + UNI EN ISO 11885:2009	< 5	< 5	< 5	< 5	< 5	< 5	< 5	< 5	< 5	< 5	< 5	< 5	< 5	< 5	< 5	< 5	< 5	< 5	< 5	< 5
	Lithium	7439-93-2	mg/kg	UNI EN 13657:2004 + UNI EN ISO 11885:2009	< 5	< 5	< 5	< 5	< 5	< 5	< 5	< 5	< 5	5.77	< 5	< 5	< 5	< 5	< 5	< 5	< 5	< 5	< 5	< 5
	Selenium	7782-49-2	mg/kg	UNI EN 13657:2004 + UNI EN ISO 11885:2009	< 5	< 5	< 5	< 5	< 5	< 5	< 5	< 5	< 5	< 5	< 5	< 5	< 5	< 5	< 5	< 5	< 5	< 5	< 5	< 5
	Magnesium	7439-95-4	mg/kg	UNI EN 13657:2004 + UNI EN ISO 11885:2009	151	149	167	157	157	163	143	164	159	163	130	146	148	172	134	148	163	155	161	149
	Tin	7440-31-5	mg/kg	UNI EN 13657:2004 + UNI EN ISO 11885:2009	< 5	< 5	< 5	< 5	< 5	< 5	< 5	< 5	< 5	< 5	< 5	< 5	< 5	< 5	< 5	< 5	< 5	< 5	< 5	< 5
	Iron	7439-89-6	mg/kg	UNI EN 13657:2004 + UNI EN ISO 11885:2009	138	113	144	129	122	133	119	121	134	109	126	125	132	136	128	133	136	128	110	140
	Copper	7440-50-8	mg/kg	UNI EN 13657:2004 + UNI EN ISO 11885:2009	27.6	28.3	31.7	29.1	28.2	32.5	29.7	33.4	30.4	30.7	29.5	26.7	29.9	29.3	38.4	31.8	29.7	32.8	29.5	29
	Titanium	7440-32-6	mg/kg	UNI EN 13657:2004 + UNI EN ISO 11885:2009	< 5	< 5	< 5	< 5	< 5	< 5	< 5	< 5	< 5	< 5	< 5	< 5	< 5	< 5	< 5	< 5	6.94	< 5	< 5	< 5
	boron	7440-42-8	mg/kg	UNI EN 13657:2004 + UNI EN ISO 11885:2009	6.12	8.81	5.78	5.04	< 5	5.69	5.85	7.71	< 5	5.77	< 5	< 5	6.14	6.46	< 5	6.24	5.46	5.75	5.34	< 5
	Barium	7440-39-3	mg/kg	UNI EN 13657:2004 + UNI EN ISO 11885:2009	< 5	< 5	< 5	< 5	< 5	< 5	< 5	< 5	< 5	5.77	< 5	< 5	< 5	< 5	< 5	< 5	< 5	< 5	< 5	< 5
	Rubidium	7440-17-7	mg/kg	UNI EN 13657:2004 + UNI EN ISO 11885:2009	5.99	< 5	< 5	< 5	5.9	< 5	6.08	5.69	5.37	5.84	5.71	5.41	< 5	< 5	< 5	5.09	5.15	< 5	< 5	< 5
	Strontium	7440-24-6	mg/kg	UNI EN 13657:2004 + UNI EN ISO 11885:2009	< 5	< 5	< 5	< 5	< 5	< 5	< 5	< 5	< 5	5.04	< 5	< 5	< 5	< 5	< 5	< 5	< 5	< 5	< 5	< 5
	Manganese	7439-96-5	mg/kg	UNI EN 13657:2004 + UNI EN ISO 11885:2009	< 5	< 5	< 5	< 5	< 5	< 5	< 5	< 5	< 5	5.77	< 5	< 5	< 5	< 5	< 5	< 5	< 5	< 5	< 5	< 5
	Molybdenum	7439-98-7	mg/kg	UNI EN 13657:2004 + UNI EN ISO 11885:2009	< 5	< 5	< 5	< 5	< 5	< 5	< 5	< 5	< 5	5.77	< 5	< 5	< 5	< 5	< 5	< 5	< 5	< 5	< 5	< 5
	Vanadium	7440-62-2	mg/kg	UNI EN 13657:2004 + UNI EN ISO 11885:2009	< 5	< 5	< 5	< 5	< 5	< 5	< 5	< 5	< 5	< 5	< 5	< 5	< 5	< 5	< 5	< 5	< 5	< 5	< 5	< 5
	Antimony	7440-36-0	mg/kg	UNI EN 13657:2004 + UNI EN ISO 11885:2009	< 5	< 5	< 5	< 5	< 5	< 5	< 5	< 5	< 5	< 5	< 5	< 5	< 5	< 5	< 5	< 5	< 5	< 5	< 5	< 5
	Chromium	7440-47-3	mg/kg	UNI EN 13657:2004 + UNI EN ISO 11885:2009	< 5	< 5	< 5	< 5	< 5	< 5	< 5	< 5	< 5	< 5	< 5	< 5	< 5	< 5	< 5	< 5	< 5	< 5	< 5	< 5
	Tungsten sulfide/stearte	12138-09-9	mg/kg	UNI EN 13657:2004 + UNI EN ISO 11885:2009	57.5	55.3	56.9	60.5	57	53.7	54.5	59.5	51.3	58.9	57.6	48.9	57.9	56.7	52.7	53.9	58.6	57.2	58.9	54.7
	CrIII	16065-83-1	mg/kg	UNI EN 13657:2004 + UNI EN ISO 11885:2009	< 5	< 5	< 5	< 5	< 5	< 5	< 5	< 5	< 5	< 5	< 5	< 5	< 5	< 5	< 5	< 5	< 5	< 5	< 5	< 5
	Zinc oxide (calculated on the basis of Zn value)	1314-13-2	mg/kg	UNI EN 13657:2004 + UNI EN ISO 11885:2009	12336	11826	12960	11933	12462	13415	12511	13085	12044	12541	12250	11716	12104	12711	12042	11757	13607	12362	12049	12354
	Titanium oxide (calculated on the basis of Ti value)	13463-67-7	mg/kg	UNI EN 13657:2004 + UNI EN ISO 11885:2009	< 10	< 10	< 10	< 10	< 10	< 10	< 10	< 10	< 10	< 10	< 10	< 10	< 10	< 10	< 10	< 10	< 10	< 10	< 10	< 10
	Magnesium oxide (calculated on the basis of Mn value)	1309-48-4	mg/kg	UNI EN 13657:2004 + UNI EN ISO 11885:2009	252	248	278	262	262	272	238	273	265	272	217	243	247	287	223	247	272	258	268	248
	CrVI	18540-29-9	mg/kg	UNI EN 13657:2004 + UNI EN ISO 11885:2009	< 5	< 5	< 5	< 5	< 5	< 5	< 5	< 5	< 5	< 5	< 5	< 5	< 5	< 5	< 5	< 5	< 5	< 5	< 5	< 5
	2-Mercaptobenzothiazole	149-30-4	mg/kg	EN 14350-2	88.4	85.1	77.4	90.9	97.6	123.0	302.0	133.5	105.2	70.4	86.0	88.8	49.8	78.1	128.3	35.6	44.2	38.4	83.7	38.9
	2,2'-Dithiobis(benzothiazole)	120-78-5	mg/kg	EN 14350-2	< 5	< 5	< 5	< 5	< 5	< 5	< 5	< 5	< 5	< 5	< 5	< 5	< 5	< 5	< 5	< 5	< 5	< 5	< 5	< 5
	N-Cyclohexyl-2-benzothiazolesulfenamide (CBS)	95-33-0	mg/kg	EN 14350-2	< 5	< 5	< 5	< 5	< 5	< 5	< 5	< 5	< 5	< 5	< 5	< 5	< 5	< 5	< 5	< 5	< 5	< 5	< 5	< 5
benzothiazoles	N-cyclohexyl-1,3-benzothiazol-2-amine	28291-75-0	mg/kg	EN 14350-2	8.3	5.4	5.3	8.1	15.6	14.5	23.0	13.9	17.1	10.7	21.6	19.8	4.2	24.3	16.5	9.1	14.1	11.8	19.9	7.9
	Benzothiazole	95-16-9	mg/kg	EN 14350-2	35.3	38.4	34.9	43.7	43.5	46.8	43.6	47.4	51.4	41.0	44.3	36.2	35.7	37.8	38.7	40.3	46.8	42.5	29.5	40.6
	2-Aminobenzothiazole	136-95-8	mg/kg	EN 14350-2	< 5	< 5	< 5	< 5	< 5	< 5	< 5	< 5	< 5	< 5	< 5	< 5	< 5	< 5	< 5	< 5	< 5	< 5	< 5	< 5
	Benzothiazole, 2-(methylthio)	615-22-5	mg/kg	EN 14350-2	1.66	1.59	1.36	1.34	1.22	1.43	1.64	2.00	3.99	1.46	1.51	1.63	0.74	1.40	2.87	1.56	2.33	2.23	8.30	1.45
	Benzothiazolone (2-Hydroxybenzothiazole)	934-34-9	mg/kg	EN 14350-2	62.6	60.4	52.1	54.6	65.6	77.9	43.4	59.9	74.5	53.4	61.6	49.5	39.7	54.8	44.0	59.2	54.6	58.2	33.2	44.3
	Bisphenol A	80-05-7	mg/kg	UNI CEN/TS 13130-13:2006	< 5	< 5	< 5	< 5	< 5	< 5	< 5	< 5	< 5	< 5	< 5	< 5	< 5	< 5	< 5	< 5	< 5	< 5	< 5	< 5
	4-t-octylphenol	140-66-9	mg/kg	EPA 3550C 2007 + EPA 8270E 2017	31.7	36.2	22.1	40.2	26.7	29.3	28.8	42.4	32.3	37.6	39.8	31.3	13.9	24.7	37.0	42.7	39.0	43.7	28.2	29.2
phenols	4-tert butylphenol	98-54-4	mg/kg	EPA 3550C 2007 + EPA 8270E 2017	3.58	2.97	4.27	3.84	1.58	1.62	0.90	3.16	1.50	3.45	2.52	3.69	1.28	4.44	1.63	1.59	7.73	3.23	0.89	3.53
	Phenol	108-95-2	mg/kg	EPA 3550C 2007 + EPA 8270E 2017	< 1	< 1	< 1	< 1	< 1	< 1	< 1	< 1	< 1	< 1	< 1	< 1	< 1	< 1	< 1	< 1	< 1	< 1	< 1	< 1
	Butylated hydroxytoluene	128-37-0	mg/kg	EPA 3550C 2007 + EPA 8270E 2017	1.71	1.53	2.11	2.16	1.75	1.84	0.89	2.55	1.07	1.62	2.02	1.67	0.36	1.81	14.24	1.69	1.59	1.78	1.36	1.60
	Iso-nonylphenol	11066-49-2	mg/kg	EPA 3550C 2007 + EPA 8270E 2017	< 5	< 5	< 5	< 5	< 5	< 5	< 5	< 5	< 5	5.77	< 5	< 5	< 5	< 5	< 5	< 5	< 5	< 5	< 5	< 5
	Diisobutyl phthalate	84-69-5	mg/kg	EPA 3550C 2007 + EPA 8270E 2017	3.22	3.26	3.18	6.11	1.24	1.37	< 1	4.31	1.64	3.69	2.51	2.1	1.54	1.32	3.16	7.17	< 1	3.28	2.06	< 1
	Benzylbutyl phthalate	85-68-7	mg/kg	EPA 3550C 2007 + EPA 8270E 2017	< 1	< 1	< 1	< 1	< 1	< 1	< 1	< 1	< 1	< 1	< 1	< 1	< 1	< 1	< 1	< 1	< 1	< 1	< 1	< 1
	Bis(2-ethylhexyl) phthalate	117-81-7	mg/kg	EPA 3550C 2007 + EPA 8270E 2017	15.6	16.9	10.8	11.8	12.8	13.1	6	17.5	10.5	10.2	14.2	10.5	44.9	8.52	24.2	26.6	26.7	15.9	21.6	11.4
	Dibutyl phthalate	84-74-2	mg/kg	EPA 3550C 2007 + EPA 8270E 2017	1.36	< 1	1.3	< 1	< 1	< 1	< 1	1.33	< 1	< 1	1.00	< 1	< 1	< 1	< 1	2.33	< 1	< 1	1.05	1.11
	Dipentyl phthalate	131-18-0	mg/kg	EPA 3550C 2007 + EPA 8270E 2017	< 5	< 5	< 5	< 5	< 5	< 5	< 5	< 5	< 5	< 5	< 5	< 5	< 5	< 5	< 5	< 5	< 5	< 5	< 5	< 5
	Dicyclohexylphthalate (DCHP)	84-61-7	mg/kg	EPA 3550C 2007 + EPA 8270E 2017	< 5	< 5	< 5	< 5	< 5	< 5	< 5	< 5	< 5	< 5	< 5	< 5	< 5	< 5	< 5	< 5	< 5	< 5	< 5	< 5
	Diisononyl phthalate	28553-12-0	mg/kg	EPA 3550C 2007 + EPA 8270E 2017	< 10	< 10	< 10	< 10	< 10	< 10	< 10	< 10	< 10	< 10	< 10	< 10	< 10	< 10	< 10	< 10	< 10	< 10	< 10	< 10
phthalates	Di-n-octyl phthalate	117-84-0	mg/kg	EPA 3550C 2007 + EPA 8270E 2017	< 1	< 1	< 1	< 1	< 1	< 1	< 1	< 1	< 1	< 1	< 1	< 1	< 1	< 1	< 1	< 1	< 1	< 1	< 1	< 1
	Bis(8-methylnonyl) phthalate	89-16-7	mg/kg	EPA 3550C 2007 + EPA 8270E 2017	< 5	< 5	< 5	< 5	< 5	< 5	< 5	< 5	< 5	< 5	< 5	< 5	< 5	< 5	< 5	< 5	< 5	< 5	< 5	< 5
	Dinonyl phthalate	84-76-4	mg/kg	EPA 3550C 2007 + EPA 8270E 2017	< 5	< 5	< 5	< 5	< 5	< 5	< 5	< 5	< 5	5.77	< 5	< 5	< 5	< 5	< 5	< 5	< 5	< 5	< 5	< 5
	Diphenyl phthalate	84-62-8	mg/kg	EPA 3550C 2007 + EPA 8270E 2017	< 5	< 5	< 5	< 5	< 5	< 5	< 5	< 5	< 5	< 5	< 5	< 5	< 5	< 5	< 5	< 5	< 5	< 5	< 5	< 5
	Diethyl phthalate	84-66-2	mg/kg	EPA 3550C 2007 + EPA 8270E 2017	< 1	< 1	< 1	< 1	< 1	< 1	< 1	< 1	< 1	< 1	< 1	< 1	< 1	< 1	< 1	< 1	< 1	< 1	< 1	< 1
	Dimethyl phthalate	131-11-3	mg/kg	EPA 3550C 2007 + EPA 8270E 2017	< 1	< 1	< 1	< 1	< 1	< 1	< 1	< 1	< 1	< 1	< 1	< 1	< 1	< 1	< 1	< 1	< 1	< 1	< 1	< 1
	Bis(2-ethylhexyl) adipate	103-23-1	mg/kg	EPA 3550C 2007 + EPA 8270E 2017	28.4	21.9	49.2	22.3	57.9	51.5	14.6	30.2	50.9	18.4	39.8	27.2	50.2	89.5	20.6	11.5	71.0	29.6	16.1	22.7
	Di-''isodecyl'' phthalate	26761-40-0	mg/kg	EPA 3550C 2007 + EPA 8270E 2017	< 10	< 10	< 10	< 10	< 10	< 10	< 10	< 10	< 10	< 10	< 10	< 10	< 10	< 10	< 10	< 10	< 10	< 10	< 10	< 10
	PCB 153-2,2',4,4',5,5'-Hexachlorobiphenyl	35065-27-1	mg/kg	EPA 3550C 2007 + EPA 8270E 2017	< 1	< 1	< 1	< 1	< 1	< 1	< 1	< 1	< 1	< 1	< 1	< 1	< 1	< 1	< 1	< 1	< 1	< 1	< 1	< 1
	PCB 101- 2,2',4,5,5'-Pentachlorobiphenyl	37680-73-2	mg/kg	EPA 3550C 2007 + EPA 8270E 2017	< 1	< 1	< 1	< 1	< 1	< 1	< 1	< 1	< 1	< 1	< 1	< 1	< 1	< 1	< 1	< 1	< 1	< 1	< 1	< 1
PCBs	PCB 138- 2,2',3,4,4',5'-Hexachlorobipheny	35065-28-2	mg/kg	EPA 3550C 2007 + EPA 8270E 2017	< 1	< 1	< 1	< 1	< 1	< 1	< 1	< 1	< 1	< 1	< 1	< 1	< 1	< 1	< 1	< 1	< 1	< 1	< 1	< 1
	PCB 180- 2,2',3,4,4',5,5'-Heptachlorobipheny	35065-29-3	mg/kg	EPA 3550C 2007 + EPA 8270E 2017	< 1	< 1	< 1	< 1	< 1	< 1	< 1	< 1	< 1	< 1	< 1	< 1	< 1	< 1	< 1	< 1	< 1	< 1	< 1	< 1
	PCB 28- 2,4,4'-trichlorobiphenyl	7012-37-5	mg/kg	EPA 3550C 2007 + EPA 8270E 2017	< 1	< 1	< 1	< 1	< 1	< 1	< 1	< 1	< 1	< 1	< 1	< 1	< 1	< 1	< 1	< 1	< 1	< 1	< 1	< 1
	Distillates (petroleum), hydrotreated heavy naphthenic ***	64742-52-5																						
	Distillates (petroleum), hydrotreated heavy paraffinic ***	64742-54-7																						
	Distillates (petroleum), solvent-dewaxed heavy paraffinic ***	64742-65-0																						
	Distillates (petroleum), solvent-refined heavy paraffinic ***	64741-88-4																						
hydrocarbon oils	Extracts (petroleum), solvent-refined heavy paraffinic distillate solvent ***	68783-04-0	mg/kg (as total hydrocarbons)	EPA 3550C 2007 + EPA 8015C 2007	34426	33449	24110	30062	23054	21517	12556	32485	20720	36200	37360	29141	22141	19345	47931	46923	20539	40428	61032	30242
	Petroleum Naphtha ***	8030-30-6																						
	White gasoline ***	8006-61-9																						
	(E)-But-2-ene (polyburadiene) ***	9003-17-2																						
	Paraffin waxes and Hydrocarbon waxes, microcryst.(antilux 654) ***	63231-60-7																						
	Aniline	62-53-3	mg/kg	EPA 3550C 2007 + EPA 8270E 2017	23.7	20.7	7.8	15.9	7.04	9.51	7.01	21.1	7.96	17.9	18.2	10.4	6.2	5.78	22.2	26.6	8.39	25.2	78.4	12.3
	Nitrosodimethylamine (n-)	62-75-9	mg/kg	EPA 8321	< 5	< 5	< 5	< 5	< 5	< 5	< 5	< 5	< 5	< 5	< 5	< 5	< 5	< 5	< 5	< 5	< 5	< 5	< 5	< 5
	Nitrosodipropylamine (n-)	621-64-7	mg/kg	EPA 8321	< 5	< 5	< 5	< 5	< 5	< 5	< 5	< 5	< 5	< 5	< 5	< 5	< 5	< 5	< 5	< 5	< 5	< 5	< 5	< 5
	Cyclohexanamine	108-91-8	mg/kg	EPA 8321	< 5	< 5	< 5	< 5	< 5	< 5	< 5	< 5	< 5	24.4	41.7	< 5	< 5	< 5	< 5	27.0	21.2	30.7	< 5	6.86
amine-nitroamines	Cyclohexanamine, N-cyclohexyl-	101-83-7	mg/kg	EPA 8321	117.4	129.9	113.4	152.8	130.4	151.4	175.6	157.8	111.5	114.4	154.4	100.2	129.8	141.1	83.2	76.9	118.4	108.4	52.9	104.4
	Nitrosodiethylamine (n-)	55-18-5	mg/kg	EPA 8321	< 5	< 5	< 5	< 5	< 5	< 5	< 5	< 5	< 5	< 5	< 5	< 5	< 5	< 5	< 5	< 5	< 5	< 5	< 5	< 5
	Nitrosopyrrolidine (n-)	930-55-2	mg/kg	EPA 8321	< 5	< 5	< 5	< 5	< 5	< 5	< 5	< 5	< 5	< 5	< 5	< 5	< 5	< 5	< 5	< 5	< 5	< 5	< 5	< 5
	Bis-(2,2,6,6-tetramethyl-4-piperidinyl)sebacate	52829-07-9	mg/kg	EPA 8321	< 5	< 5	< 5	< 5	< 5	< 5	< 5	< 5	< 5	< 5	< 5	< 5	< 5	< 5	< 5	< 5	< 5	< 5	< 5	< 5
	tertbutylamine	75-64-9	mg/kg	EPA 8321	28.8	5.97	8.95	9.85	7.54	7.26	7.1	11.6	5.47	11.5	14.4	9.48	8.37	7.34	8.43	7.45	8.5	13	10.7	6.04
	N-(1,3-dimethylbutyl)-N'-phenyl-p-phenylenediamine (6-ppd)	793-24-8	mg/kg	EPA 8321	857.0	919.5	775.1	924.8	924.9	1059.7	1629.0	1258.1	840.2	954.4	1072.2	858.4	624.8	914.2	1265.4	921.0	1106.6	1037.1	1138.7	886.7
	1,2-Dichloroethane	107-06-2	mg/kg	EPA 3550C 2007 + EPA 8260D 2017	< 1	< 1	< 1	< 1	< 1	< 1	< 1	< 1	< 1	< 1	< 1	< 1	< 1	< 1	< 1	< 1	< 1	< 1	< 1	< 1
	Trichloroethylene	79-01-6	mg/kg	EPA 3550C 2007 + EPA 8260D 2017	< 1	< 1	< 1	< 1	< 1	< 1	< 1	< 1	< 1	< 1	< 1	< 1	< 1	< 1	< 1	< 1	< 1	< 1	< 1	< 1
	Benzene	71-43-2	mg/kg	EPA 3550C 2007 + EPA 8260D 2017	< 1	< 1	< 1	< 1	< 1	< 1	< 1	< 1	< 1	< 1	< 1	< 1	< 1	< 1	< 1	< 1	< 1	< 1	< 1	< 1
halogenated organic aromatic compounds	Styrene	100-42-5	mg/kg	EPA 3550C 2007 + EPA 8260D 2017	< 1	< 1	< 1	< 1	< 1	< 1	< 1	< 1	< 1	< 1	< 1	< 1	< 1	< 1	< 1	< 1	< 1	< 1	< 1	< 1
	Toluene	108-88-3	mg/kg	EPA 3550C 2007 + EPA 8260D 2017	< 1	< 1	< 1	< 1	< 1	< 1	< 1	< 1	< 1	< 1	< 1	< 1	< 1	< 1	< 1	< 1	< 1	< 1	< 1	< 1
	o-Xylene	95-47-6	mg/kg	EPA 3550C 2007 + EPA 8260D 2017	< 1	< 1	< 1	< 1	< 1	< 1	< 1	< 1	< 1	< 1	< 1	< 1	< 1	< 1	< 1	< 1	< 1	< 1	< 1	< 1
	1,2-Dichloropropane	78-87-5	mg/kg	EPA 3550C 2007 + EPA 8260D 2017	< 1	< 1	< 1	< 1	< 1	< 1	< 1	< 1	< 1	< 1	< 1	< 1	< 1	< 1	< 1	< 1	< 1	< 1	< 1	< 1
	Carbon Tetrachloride	56-23-5	mg/kg	EPA 3550C 2007 + EPA 8260D 2017	< 1	< 1	< 1	< 1	< 1	< 1	< 1	< 1	< 1	< 1	< 1	< 1	< 1	< 1	< 1	< 1	< 1	< 1	< 1	< 1
	p,m-xylene	179601-23-1	mg/kg	EPA 3550C 2007 + EPA 8260D 2017	< 1	< 1	< 1	< 1	< 1	< 1	< 1	< 1	< 1	< 1	< 1	< 1	< 1	< 1	< 1	< 1	< 1	< 1	< 1	< 1
	2-Pyrrolidinone. 1-methyl-	872-50-4	mg/kg	EPA 3550C 2007 + EPA 8260D 2017	< 5	< 5	< 5	< 5	< 5	< 5	< 5	< 5	< 5	< 5	< 5	< 5	< 5	< 5	< 5	< 5	< 5	< 5	< 5	< 5
	4-Methyl-2-pentanone		mg/kg	EPA 3550C 2007 + EPA 8260D 2017	16.6	19.8	23.5	21.5	23.1	51.4	56.1	24.5	20.9	21.4	14.7	21.3	17.0	33.0	23.0	20.2	20.9	15.1	15.3	22.1
	Formaldehyde	50-00-0	mg/kg	EPA 8315 A	1.67	2.41	3.2	4.49	3.07	2.03	1.92	3.52	2.95	3.26	5.7	3.43	4.3	6.03	5.46	3.79	6.44	5.21	3.53	4.63
	Butoxyethoxyethanol	112-34-5	mg/kg	EPA 3550C 2007 + EPA 8260D 2017	< 1	< 1	< 1	< 1	< 1	< 1	< 1	< 1	< 1	< 1	< 1	< 1	< 1	< 1	< 1	< 1	< 1	< 1	< 1	< 1
	Carbon Disulfide	75-15-0	mg/kg	EPA 3550C 2007 + EPA 8260D 2017	< 1	< 1	< 1	< 1	< 1	< 1	< 1	< 1	< 1	< 1	< 1	< 1	< 1	< 1	< 1	< 1	< 1	< 1	< 1	< 1
	N,N'-Diphenylguanidine (DPG)	102-06-7	mg/kg	EPA 8321	< 5	< 5	< 5	< 5	< 5	< 5	< 5	< 5	< 5	5.77	< 5	< 5	< 5	< 5	< 5	< 5	< 5	< 5	< 5	< 5
	Carbon Black	1333-86-4	%	ASTM D297-15	32.0	30.8	31.9	28.9	29.9	31.0	30.6	31.7	33.5	29.0	31.7	31.3	30.3	32.3	28.3	30.7	32.6	29.7	22.3	30.6
	Total sulfur	7704-34-9	%	ASTM D297-15	2.43	2.22	2.07	1.87	1.78	1.58	1.80	1.95	2.05	1.29	1.47	2.49	1.58	1.85	2.04	1.64	1.31	1.48	1.41	1.65
	Ethylene thiourea (Ethylene thiourea)	96-45-7	mg/kg	EPA 3550C 2007 + EPA 8270E 2017	< 5	< 5	< 5	< 5	< 5	< 5	< 5	< 5	< 5	< 5	< 5	< 5	< 5	< 5	< 5	< 5	< 5	< 5	< 5	< 5
	Acetonitrile	75-05-8	mg/kg	EPA 3550C 2007 + EPA 8260D 2017	< 1	< 1	< 1	< 1	< 1	< 1	< 1	< 1	< 1	< 1	< 1	< 1	< 1	< 1	< 1	< 1	< 1	< 1	< 1	< 1
	Azobenzene	103-33-3	mg/kg	EPA 3550C 2007 + EPA 8270E 2017	< 5	< 5	< 5	< 5	< 5	< 5	< 5	< 5	< 5	< 5	< 5	< 5	< 5	< 5	< 5	< 5	< 5	< 5	< 5	< 5
	4-nitroquinoline N-oxide	56-57-5	mg/kg	EPA 3550C 2007 + EPA 8270E 2017	< 5	< 5	< 5	< 5	< 5	< 5	< 5	< 5	< 5	< 5	< 5	< 5	< 5	< 5	< 5	< 5	< 5	< 5	< 5	< 5
	Ethanol	64-17-5	mg/kg	EPA 3550C 2007 + EPA 8015C 2007	< 5	< 5	< 5	< 5	< 5	< 5	< 5	< 5	< 5	< 5	< 5	< 5	< 5	< 5	< 5	< 5	< 5	< 5	15.1	< 5
	Methyl Alcohol	67-56-1	mg/kg	EPA 3550C 2007 + EPA 8015C 2007	< 5	< 5	< 5	< 5	< 5	< 5	< 5	< 5	< 5	< 5	< 5	< 5	< 5	< 5	< 5	< 5	< 5	< 5	< 5	< 5
	Tetraethylthiuram disulfide	97-77-8	mg/kg	EPA 3550C 2007 + EPA 8270E 2017	< 5	< 5	< 5	< 5	< 5	< 5	< 5	< 5	< 5	< 5	< 5	< 5	< 5	< 5	< 5	< 5	< 5	< 5	< 5	< 5
	Tetramethylthiuram disulfide TMTD	137-26-8	mg/kg	EPA 3550C 2007 + EPA 8270E 2017	< 5	< 5	< 5	< 5	< 5	< 5	< 5	< 5	< 5	< 5	< 5	< 5	< 5	< 5	< 5	< 5	< 5	< 5	< 5	< 5
	Di-ortho-tolylguanidine	97-39-2	mg/kg	EPA 3550C 2007 + EPA 8270D 2017	< 5	< 5	< 5	< 5	< 5	< 5	< 5	< 5	< 5	< 5	< 5	< 5	< 5	< 5	< 5	< 5	< 5	< 5	< 5	< 5
others	Bromodichloromethane	75-27-4	mg/kg	EPA 3550C 2007 + EPA 8260D 2017	< 1	< 1	< 1	< 1	< 1	< 1	< 1	< 1	< 1	< 1	< 1	< 1	< 1	< 1	< 1	< 1	< 1	< 1	< 1	< 1
	N-Oxydiethylenedithiocarbamyl-N`-oxydiethylenesulfenamide (OTOS)	13752-51-7	mg/kg	EPA 8321	< 100	< 100	< 100	< 100	< 100	< 100	< 100	< 100	< 100	< 100	< 100	< 100	< 100	< 100	< 100	< 100	< 100	< 100	< 100	< 100
	Dibenzothiophene	132-65-0	mg/kg	EPA 3550C 2007 + EPA 8260D 2017	< 5	< 5	< 5	< 5	< 5	< 5	< 5	< 5	< 5	< 5	< 5	< 5	< 5	< 5	< 5	< 5	< 5	< 5	< 5	< 5
	Benzene, 1,3-bis(1-methylethenyl)-	3748-13-8	mg/kg	EPA 3550C 2007 + EPA 8260D 2017	< 5	< 5	< 5	< 5	< 5	< 5	< 5	< 5	< 5	< 5	< 5	< 5	< 5	< 5	< 5	< 5	< 5	< 5	< 5	< 5
	Heptanonitrile	629-08-3	mg/kg	EPA 3550C 2007 + EPA 8260D 2017	< 5	< 5	< 5	< 5	< 5	< 5	< 5	< 5	< 5	< 5	< 5	< 5	< 5	< 5	< 5	< 5	< 5	< 5	< 5	< 5
	Tertbutylacetophenone	31366-07-1	mg/kg	EPA 3550C 2007 + EPA 8260D 2017	< 100	< 100	< 100	< 100	< 100	< 100	< 100	< 100	< 100	< 100	< 100	< 100	< 100	< 100	< 100	< 100	< 100	< 100	< 100	< 100
	Nonanale	124-19-6	mg/kg	EPA 3550C 2007 + EPA 8260D 2017	< 5	< 5	< 5	< 5	< 5	< 5	< 5	< 5	< 5	< 5	< 5	< 5	< 5	< 5	< 5	< 5	< 5	< 5	< 5	< 5
	Silicon dioxide (calculated on the basis of Si)	112926-00-8	%	UNI EN 15309:2007	1.55	1.94	1.16	1.14	1.04	1.60	1.11	2.14	1.28	1.71	0.71	1.08	2.43	1.77	1.02	1.49	7.18	1.39	7.18	1.39
	1,3-Butadiene/styrene copolymers	9003-55-8	mg/kg	EPA 3550C 2007 + EPA 8260D 2017	< 50	< 50	< 50	< 50	< 50	< 50	< 50	< 50	< 50	< 50	< 50	< 50	< 50	< 50	< 50	< 50	< 50	< 50	< 50	< 50
	Fatty acids, tallow, hydrogenated	61790-38-3	mg/kg	EPA 3550C 2007 + EPA 8015C 2007	< 100	< 100	< 100	< 100	< 100	< 100	< 100	< 100	< 100	< 100	< 100	< 100	< 100	< 100	< 100	< 100	< 100	< 100	< 100	< 100
	Fatty acids, C16-18	67701-03-5	mg/kg	EPA 3550C 2007 + EPA 8015C 2007	4246	3486	2271	4070	3764	2159	1473	1222	1148	1719	1171	2529	2739	2615	2280	2079	1909	1832	5115	2429
	Silica (VULKASIL S) evaluated as total Si	7631-86-9	%	UNI EN 15309:2007	1.2	1.2	0.7	0.9	0.5	0.5	0.5	0.7	0.5	1.0	0.6	0.8	0.3	0.5	1.1	0.8	0.5	0.7	3.3	0.6
	Benzene, 1,4-bis(1-methylethenyl)-	1605-18-1	mg/kg	EPA 3550C 2007 + EPA 8015C 2007	< 5	< 5	< 5	< 5	< 5	< 5	< 5	< 5	< 5	< 5	< 5	< 5	< 5	< 5	< 5	< 5	< 5	< 5	< 5	< 5
	Stearic acid	57-11-4	mg/kg	EPA 3550C 2007 + EPA 8015C 2007	1946	1863	1213	2111	1995	1205	907	622	773	951	605	1292	1494	1496	1186	1144	869	905	2141	1234
	Trimethyl-1,2-dihydroquinoline (TMDQ)	26780-96-1	mg/kg	EPA 8321	33.4	26.2	23.3	22.0	16.0	16.0	24.8	27.0	18.7	23.4	21.3	24.9	2.7	21.0	34.0	20.1	18.4	26.3	16.3	20.6
	Dimethyldiphenylthioperoxydicarbamic acid	53880-86-7	mg/kg	EPA 8321	< 100	< 100	< 100	< 100	< 100	< 100	< 100	< 100	< 100	< 100	< 100	< 100	< 100	< 100	< 100	< 100	< 100	< 100	< 100	< 100
	N-(cyclohexylthio)phthalimide	17796-82-6	mg/kg	EPA 8321	< 100	< 100	< 100	< 100	< 100	< 100	< 100	< 100	< 100	< 100	< 100	< 100	< 100	< 100	< 100	< 100	< 100	< 100	< 100	< 100
	N-tert-Butyl-2-benzothiazolesulfenamide (TBBS)	95-31-8	mg/kg	EPA 8321	< 5	< 5	< 5	< 5	< 5	< 5	< 5	< 5	< 5	5.77	< 5	< 5	< 5	< 5	< 5	< 5	< 5	< 5	< 5	< 5
	polymer content		%	ASTM D297-15	45.6	49.6	48.3	48.2	53.7	54.5	51.4	47.8	49.8	49.7	44.1	50.2	54.3	50.6	43.8	43.3	48.1	49.3	36.2	50.8
	ashes		%	ASTM D297-15	8.65	8.18	6.84	9.56	6.35	6.21	5.74	8.85	8.27	10.08	7.87	8.00	5.70	7.97	10.42	9.43	6.36	9.15	20.83	7.19

Table S1. Chemicals (mg/Kg) quantified in the 20 different ELT-dg samples (solid fraction). Zinc oxide, Titanium oxide and Magnesium oxide were not detected analytically in the samples and for this reason their presence in the ELT-dg was not certified.
