# Supplementary material for: Ecological Impact of End-of-Life-Tire (ELT)-Derived Rubbers: Acute and Chronic Effects at Organism and Population Levels
Source: Toxics. 2022 Apr 19;10(5):201. doi: 10.3390/toxics10050201 (PMC9144162; doi:10.3390/toxics10050201)
Supplement: Supplementary file 1 [file toxics-10-00201-s001.zip › Table S2.pdf]

Substance	CAS	Method	Environmental hazard classification CLP (Annex VI): hazardous to the aquatic environment	(A) mg component/kg (max) = µg/g	(B) µg component in 100 mg sample	(C) µg component/L in 100 mg sample/L suspension	(D) µg component/L in 100 mg sample/L suspension
Benzo(a)pyrene	50-32-8	EPA 3510C 1996 + EPA 8270E 2018	H400 Aquatic acute 1<br>H410 Aquatic chronic 1	1.6	0.2	0.2	< 0.0011
Benzo(a)anthracene	56-55-3	EPA 3510C 1996 + EPA 8270E 2018	H400 Aquatic acute 1 M=100<br>H410 Aquatic chronic 1 M=100	1.6	0.2	0.2	< 0.0011
Benzo(b)fluoranthene	205-99-2	EPA 3510C 1996 + EPA 8270E 2018	H400 Aquatic acute 1<br>H410 Aquatic chronic 1	0.9	0.1	0.1	< 0.0012
Benzo(e)pyrene	192-97-2	EPA 3510C 1996 + EPA 8270E 2018	H400 Aquatic acute 1<br>H410 Aquatic chronic 1	1.7	0.2	0.2	< 0.0010
Chrysene	218-01-9	EPA 3510C 1996 + EPA 8270E 2018	H400 Aquatic acute 1<br>H410 Aquatic chronic 1	1.8	0.2	0.2	< 0.0011
Naphthalene	91-20-3	EPA 3510C 1996 + EPA 8270E 2018	H400 Aquatic acute 1<br>H410 Aquatic chronic 1	1.1	0.1	0.1	0.0101
Lead	7439-92-1	EPA 6020B 2014	H400 Aquatic acute 1<br>H410 Aquatic chronic 1	14.6	1.5	1.5	< 0.61
Cobalt	7440-48-4	EPA 6020B 2014	H413 Aquatic chronic 4	153.0	15.3	15.3	< 0.31
Cobalt sulfide	1317-42-6	P-AM-817 Rev.0	H400 Aquatic acute 1 M=100<br>H410 Aquatic chronic 1 M=100	-	-	-	< 0.50

Zinc	7440-66-6	EPA 6020B 2014	H400 Aquatic acute 1<br>H410 Aquatic chronic 1	10919.0	1091.9	1091.9	53.4
Arsenic	7440-38-2	EPA 6020B 2014	H410 Aquatic chronic 1	5.8	0.6	0.6	< 0.30
Copper	7440-50-8	EPA 6020B 2014	H411 Aquatic chronic 2 proposed classification	38.4	3.8	3.8	2.23
2-mercaptobenzothiazole	149-30-4	EPA 3510C 1996 + EPA 8270E 2018	H400 Aquatic acute 1<br>H410 Aquatic chronic 1	302.0	30.2	30.2	< 1
4-t-octylphenol	140--66-9	EPA 3510C 1996 + EPA 8270E 2018	H400 Aquatic acute 1 M=10<br>H410 Aquatic chronic 1 M=10	43.7	4.4	4.4	< 1
4-tert butylphenol	98-54-4	EPA 3510C 1996 + EPA 8270E 2018	H410 Aquatic chronic 1 M=1	7.7	0.8	0.8	< 1
Diisobutyl phthalate	84-69-5	EPA 3510C 1996 + EPA 8270E 2018	H400 Aquatic acute 1	7.2	0.7	0.7	0.764
Dibutyl phtalate	84-74-2	EPA 3510C 1996 + EPA 8270E 2018	H400 Aquatic acute 1	2.3	0.2	0.2	0.186
Aniline	62-53-3	EPA 3510C 1996 + EPA 8270E 2018	H400 Aquatic acute 1	78.4	7.8	7.8	0.851
Cyclohexanamine, N-cyclohexyl-	101-83-7	EPA 3510C 1996 + EPA 8270E 2018	H400 Aquatic acute 1<br>H410 Aquatic chronic 1	175.6	17.6	17.6	< 1
N,N'-diphenylguanidine (DPG)	102-06-7	EPA 3510C 1996 + EPA 8270E 2018	H411 Aquatic chronic 2	5.8	0.6	0.6	< 1

Table S2. Twenty selected chemicals with the concentration detected in the ELT suspensions. In the table are reported the CAS number, the used methods for the chemical detection, the CLP classification as well as the theoretical and analytical concentration of considered substances/elements. In particular, in the column (A) it is reported the max concentration measured in the 100.0 mg/L of ELT-dg suspensions (mg of each component in 1 Kg of ELT-dg, corresponding to µg in 1 g of ELT-dg; Table S1), in the column (B) the µg component in 100 mg of sample (calculated form max concentration), in the column (C) the theoretical concentration calculated assuming the hypothesis that all the amount present in 100 mg of ELT-dg was dissolved/solubilized in 1 L of water and, lastly, in the column (D) the analytical concentration measured in the 100.0 mg/L of ELT-dp.
